# Direct inference of haplotypes from sequencing data

**DOI:** 10.1093/bioadv/vbaf195

**Published:** 2025-08-20

**Authors:** Zhen Zhang, Bencong Zhu, Yongyi Luo, Jiandong Shi, Sheng Lian, Jingyu Hao, Taobo Hu, Toyotaka Ishibashi, Depeng Wang, Shu Wang, Weichuan Yu, Xiaodan Fan

**Affiliations:** The First Institute, Kunming Institute of Physics, Kunming, Yunnan Province, 650223, China; Department of Electronic and Computer Engineering, The Hong Kong University of Science and Technology, Hong Kong SAR, 999077, China; Department of Statistics, The Chinese University of Hong Kong, Hong Kong SAR, 999077, China; Department of Statistics, The Chinese University of Hong Kong, Hong Kong SAR, 999077, China; Department of Statistics, The Chinese University of Hong Kong, Hong Kong SAR, 999077, China; Department of Statistics, The Chinese University of Hong Kong, Hong Kong SAR, 999077, China; Department of Statistics, The Chinese University of Hong Kong, Hong Kong SAR, 999077, China; Department of Electronic and Computer Engineering, The Hong Kong University of Science and Technology, Hong Kong SAR, 999077, China; Department of Breast Surgery, Peking University People’s Hospital, Beijing, 100044, China; Division of Life Science, Hong Kong University of Science and Technology, Hong Kong SAR, 999077, China; GrandOmics Inc, Beijing, 102200, China; Department of Breast Surgery, Peking University People’s Hospital, Beijing, 100044, China; Department of Electronic and Computer Engineering, The Hong Kong University of Science and Technology, Hong Kong SAR, 999077, China; Department of Statistics, The Chinese University of Hong Kong, Hong Kong SAR, 999077, China

## Abstract

**Motivation:**

Haplotypes are crucial for various genetic analyses, but reconstructing haplotypes from sequencing data remains a significant challenge. Current methods for haplotype reconstruction typically rely on a procedure of two separated stages, variant calling and phasing, but phasing overlooks the errors in variant calling. Additionally, the complexity of haplotype reconstruction increases with the number of homologous chromosomes in the sample, a common scenario in polyploid species or cell mixture sequencing.

**Results:**

To address the challenges above, we propose a unified probabilistic framework that directly utilizes sequencing reads to estimate haplotypes and sequencing error profiles. Rather than focusing solely on variant loci used by traditional phasing methods, our approach models all loci covered by any sequencing read to enhance the estimation of error profiles in sequencing data, thereby increasing the statistical power of haplotype inference, especially for low-coverage datasets. Evaluations on both simulated and real sequencing data demonstrate the superior performance of our method, particularly in scenarios characterized by high sequencing error rates, low coverage, or polyploidy.

**Availability and implementation:**

Related codes and dataset can be found at: https://github.com/new-zbc/DIHap.

## 1 Introduction

Haplotypes are combinations of alleles from multiple genetic loci located on the same chromosome that are inherited together. Haplotype information is fundamental for medical and population genetics, where it is used to study genetic variation associated with human diseases or therapeutic drugs (Farci *et al.* 2002, Clark, 2004). For diploid genomes, a specific segment of chromosomal DNA normally harbors two haplotypes, one inherited from each parent. But more than two haplotypes may exist at any given chromosomal region when genotyping polyploid genomes or a mixture of diploid cell populations, which is common for sequencing tumor tissues contaminated with normal cells (Garg 2021).

The reconstruction of haplotypes relies on genotyping technologies. The current mainstream genotyping technologies are next-generation sequencing (NGS) and third-generation sequencing (TGS). In contrast to NGS, TGS produces longer sequencing reads, exceeding 1000 base pairs on average. This capability allows TGS to detect the linkage of two or more single nucleotide variations (SNVs) within a single read, thus reducing the error caused by the assembly procedure and enhancing the accuracy of downstream haplotype inference. However, the drawbacks of TGS are the higher cost and higher error rate, which underscore the necessity for developing more powerful algorithms to infer haplotypes from low-coverage data.

The existing haplotype inference methods follow a two-stage procedure: first detect SNVs by variant detection algorithms, then phase the SNVs to construct haplotypes. For the first stage, *PEPPER-Margin-DeepVariant* is a commonly used SNV calling tool for TGS data (Shafin *et al.* 2021). *FreeBayes* is a haplotype-aware detection tool under a Bayesian framework (Garrison and Marth 2012). Recently, some tools have employed deep neural network techniques for variant detection. *DeepVariant* is one representative of such tools to detect small variants (Poplin *et al.* 2018). Based on *DeepVariant*, *Clair3* enhances accuracy by combining pileup calling for individual site with full-alignment calling to leverage neighboring information (Zheng *et al.* 2022). For the second stage, previously identified variants are used for phasing. Existing phasing tools can be grouped into two main categories. The first category uses graph-based community detection methods, which treat haplotype inference as the identification of read communities, exemplified by tools like *HapCUT* (Bansal and Bafna 2008), *RefHap* (Duitama *et al.* 2012), *ProbHap* (Kuleshov 2014), and *HapCut2* (Edge *et al.* 2017). The second category is based on combinatorial optimization models (Browning and Browning 2011), such as minimum error correction (MEC) score(Wang *et al.* 2005), minimum fragment removal (Lancia *et al.* 2001), minimum SNP removal (Lancia *et al.* 2001), and minimum fragment cut (Duitama *et al.* 2012). The tools within the second category search for a set of haplotypes with the highest fitness score.

Despite advancements in both stages of the aforementioned procedure, several challenges persist. Primarily, the uncertainty in variant calling is not properly propagated to the phasing stage, thus the two-stage procedure cannot provide comprehensive uncertainty quantification and the best statistical efficiency. Secondly, the possible existence of multiple haplotypes in the sequencing data due to cell impurity or polyploidy makes the inference much more challenging due to the expanded parameter space for exploration and the reduced effective sample size for each haplotype (Yang *et al.* 2017). Several phasing algorithms have been devised for polyploid cases. For instance, *HPOP* and its genotype-constrained version *HPOPG* used dynamic programming for phasing (Xie *et al.* 2016). *AltHap* allowed a SNV site to be polyallelic in polyploid cases and used the tensor decomposition to infer the haplotypes (Hashemi *et al.* 2018). *WhatsHap* used the similarity among variant segments to perform clustering, then threaded the variant segment clusters to haplotypes (Schrinner *et al.* 2020). In 2022, Shaw and Yu developed *flopp* which performed a min-sum max tree partition to infer the haplotypes instead of minimizing the MEC score (Shaw and Yu 2022). Nonetheless, a unified framework for haplotype inference directly grounded on original reads, particularly in the intricate polyploid setting, remains absent.

To address the challenges above, we propose a framework for Direct Inference of Haplotypes from sequencing data (DIHap), which constructs a probabilistic model for haplotypes and sequencing error profiles. An efficient Classification Annealing Expectation Maximization algorithm (CAEM), motivated by the work of Celeux and Govaert (1992), has been designed to estimate haplotypes and model parameters. Extensive simulations and real applications in both diploid and polyploid data demonstrate its superiority over existing methods, which are all two-stage methods. Our major contributions are as follows. First, it is the first unified probabilistic haplotype inference framework directly based on original reads instead of identified variants. Secondly, instead of only considering variant loci, all sequenced loci have been modeled to estimate the error profile of the corresponding sequencing technique, increasing statistical power of phasing for low-coverage sequencing data. Thirdly, it outperforms existing methods for challenging polyploid cases. In simulated and real polyploid TGS data, *DIHap* provides the best haplotype inference results.

The later part of the article is organized as follows. Section 2 is dedicated to the formulation of the proposed framework and the details of the computing algorithm. In Section 3, we evaluate *DIHap* on extensive simulations, including low-coverage scenarios and polyploid scenarios. We also compare the performance of *DIHap* to existing methods in the real applications to diploid and polyploid data in Section 4.1. Finally, we conclude our study in Section 5.

## 2 Methods

We introduce a generative model for the hidden haplotypes and observed sequencing reads, then design the CAEM algorithm to fit the model to the input data. The input data consists of aligned reads sequenced from a *K*-ploid organism or bulk sequencing of tumors contaminated with normal cells, along with a reference genome to which the reads are aligned. We aim to infer the *K* haplotypes.

**Figure 1.**
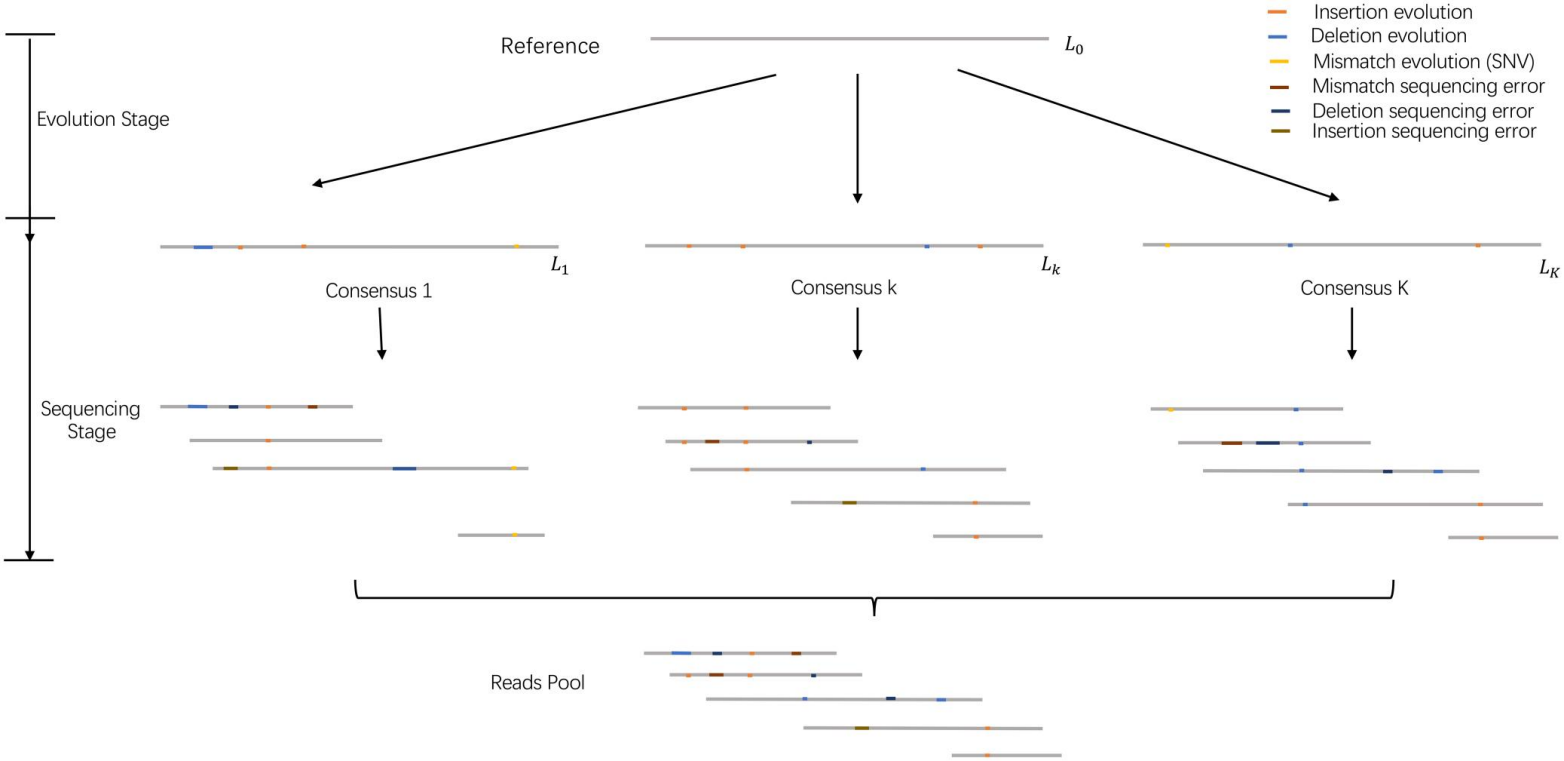
The two-stage generative model. In the evolution stage, the *K* consensus sequences are generated from the reference with the probability of substitution (thus creating a mismatch in alignment), deletion, and insertion. While in the sequencing stage, the sequencing tools randomly pick one of the consensus sequences as a template to generate the reads with possible errors following a sequencing error profile, which specifies the probabilities of mismatch, deletion and insertion errors. Finally, all the reads are aligned to the reference sequence.

Traditionally, a haplotype is a sequence of variant sites. We consider the whole genomic sequence containing this haplotype and name it a consensus sequence. The generative process of the observed reads follows an evolution stage and then a sequencing stage, as shown in Fig. 1. The evolution stage specifies how the consensus sequences independently evolved from the reference sequence, where we consider three types of mutations, substitution, insertion, and deletion. The sequencing stage specifies how sequencing errors occur between the biological truth (i.e. the consensus sequence) and the observed reads.

### 2.1 Evolution stage model

The model during the evolution stage concerns the generation of consensus sequences from the reference genome. Suppose that the reference genome *r* contains $L_{0}$ nucleotides and there are *K* consensus $C=\{C_{1},\ldots ,C_{K}\}$ independently evolved from the reference sequence with substitution, insertion, and deletion mutations. The consensus sequence $C_{k}$ consists of elements from the set B={“A”,“T”,“C”,“G”,“−”}, where “−” represents a deletion mutation compared to the reference sequence. Based on the reference sequence, the base of $C_{k}$ can be generated by modelling the positions and the gaps between bases. For each position $1\leq l\leq L_{0}$, we introduce a random variable $X_{kl}\in \{1,2,3\}$, representing no-mutation, substitution, and deletion respectively. It is defined as

$X_{kl}=1$ : $r(l)=C_{k}(l)$, the *l*th position of *r* is evolved correctly from the reference sequence.

$X_{kl}=2$ : $r(l)\neq C_{k}(l)$, the *l*th position of *r* is substituted by a different base.

$X_{kl}=3$ : $C_{k}(l)=“-”$, the *l*th position of *r* is deleted.

where $C_{k}(l)$ and r(l) are the nucleoside at the *l*th position of $C_{k}$ and the reference sequence, respectively.

The insertion mutation occurs at the gaps between neighboring bases in the reference. There are total $L_{0}-1$ gaps for the reference with $L_{0}$ bases. For each gap $1\leq g\leq L_{0}-1$, we introduce a random variable ${\tilde{X}}_{kg}$ such that ${\tilde{X}}_{kg}=1$ if a base is inserted when compared to the *k*th consensus at the *g*th gap of the reference sequence and ${\tilde{X}}_{kg}=0$ otherwise. Then the likelihood of $C_{k}$ conditional on the reference sequence *r* is

(1) $$\begin{array}{c} P(C_{k}|r)=\prod_{l=1}^{L_{0}}\gamma_{k,1}^{1(X_{kl}=1)}\gamma_{k,2}^{1(X_{kl}=2)}\gamma_{k,3}^{1(X_{kl}=3)}\\ \times \prod_{g=1}^{L_{0}-1}\beta_{k}^{1({\tilde{X}}_{kg}=1)}{\left(1-\beta_{k}\right)}^{1({\tilde{X}}_{kg}=0)} \end{array}$$

 

(2) $$\begin{array}{c} ={\left(1-\beta_{k}\right)}^{\left(L_{0}-1-N_{k}\right)}\beta_{k}^{N_{k}}\prod_{j=1}^{3}\gamma_{k,j}^{n_{k,j}}, \end{array}$$

where $n_{k,j}=\sum_{l=1}^{L_{0}}1(X_{kl}=j)$ for j∈{1,2,3} denotes the number of the positions in the *j*th scenario mentioned above, and the number of insertion mutations is $N_{k}=\sum_{g=1}^{L_{0}-1}1({\tilde{X}}_{kg}=1)$. The parameter $\left(\gamma_{k,1},\gamma_{k,2},\gamma_{k,3},\beta_{k}\right)$ represents the evolution probability for $C_{k}$, which are defined in Table 1. Note that $\sum_{j=1}^{3}\gamma_{k,j}=1$.

**Table 1.** Model parameters and related symbols.

Symbol	Meaning
$\gamma_{k,1}$	No-mutation probability per position from *r* to $C_{k}$
$\gamma_{k,2}$	Substitution probability per position from *r* to $C_{k}$
$\gamma_{k,3}$	Deletion probability per position from *r* to $C_{k}$
$\beta_{k}$	Insertion probability per gap of *r*
$p_{1}$	Correct sequencing probability per base
$p_{2}$	Mismatch sequencing error probability per base
$p_{3}$	Deletion sequencing error probability per location
$p_{4}$	Insertion sequencing error probability per location
$\lambda^{\text{ins}}$	Average length of insertion sequencing error
$\lambda^{\text{del}}$	Average length of deletion sequencing error

### 2.2 Sequencing stage model

In the sequencing stage, each read is generated based on a segment of a consensus sequence with several types of sequencing errors, including mismatch sequencing error, insertion sequencing error, and deletion sequencing error. Suppose that $R_{i}$ is the *i*th read generated from $C_{k}$, covering positions $\left[s_{i},e_{i}\right]$. The length $L_{i}=e_{i}-s_{i}+1$. A latent indicator vector $z_{i}=(z_{i,1},\ldots ,z_{i,K})$ is introduced such that only $z_{i,k}=1$ if $R_{i}$ is sequenced from the *k*th consensus. Given $z_{i,k}=1$, the alignment result between $R_{i}$ and the *k*th consensus sequence $C_{k}$ at the *l*th position is classified similarly into 3 categories. Note that $l\in [s_{i},e_{i}]$. We define a random variable $Y_{\mathrm{ikl}}\in \{1,2,3\}$ representing the following cases:

$Y_{\mathrm{ikl}}=1$ : $R_{i}(l)=C_{k}(l)$, the base at the *l*th position of $C_{k}$ is sequenced correctly.

$Y_{\mathrm{ikl}}=2$ : $R_{i}(l)\neq C_{k}(l)$, $R_{i}$ has a mismatch sequencing error at the *l*th position.

$Y_{\mathrm{ikl}}=3$ : $R_{i}(l)=“-”\;\&\;C_{k}(l)\neq “-”$, $R_{i}$ has a deletion sequencing error at the *l*th position.

A similar random variable ${\tilde{Y}}_{\mathrm{ikg}}$ can also be introduced such that ${\tilde{Y}}_{\mathrm{ikg}}=1$ represents an insertion sequencing error at the *g*th gap for $s_{i}\leq g\leq e_{i}-1$. In the sequencing stage, the deletion and insertion error may delete and insert a contiguous segment instead of a single base. To jointly model the deletion segment length $L_{i}^{\text{del}}$ and insertion segment length $L_{i}^{\text{ins}}$, we consider the truncated Poisson distribution by $L_{i}^{\text{del}}-1∼\text{Poi}(\lambda^{\text{ins}})$ and $L_{i}^{\text{del}}-1∼\text{Poi}(\lambda^{\text{del}})$. Hence the probability of read $R_{i}$ is

(3) $$\begin{array}{l} \;P(R_{i}|C_{k},\psi )\\ =\prod_{l\in [s_{i},e_{i}]}p_{1}^{1(Y_{\mathrm{ikl}}=1)}p_{2}^{1(Y_{\mathrm{ikl}}=2)} \end{array}$$

 

(4) $$\begin{array}{c} \times \prod_{u=1}^{U_{ik}}p_{3}^{1(Y_{ikl_{u}}=3)}\text{Poi}(L_{iu}^{\mathrm{del}}-1|\lambda^{\mathrm{del}}) \end{array}$$

 

(5) $$\begin{array}{c} \times {\left(1-p_{4}\right)}^{W_{ik}}\prod_{v=1}^{V_{ik}}p_{4}^{1({\tilde{Y}}_{ikg_{v}}=1)}\text{Poi}(L_{iv}^{\mathrm{ins}}-1|\lambda^{\mathrm{ins}}) \end{array}$$

where the part (4) and (5) represents the likelihood of total $U_{ik}$ deletion segments and $V_{ik}$ insertion segments when $R_{i}$ aligned to $C_{k}$. The position index $l_{u}$ represents the first position of the *u*th deletion block with length $L_{iu}^{\text{del}}$. The position index $g_{v}$ represents the $g_{v}$th gap where the *v*th insertion block with length $L_{iv}^{\text{ins}}$ is inserted. $W_{i}^{k}$ represents the number of gaps without insertion segment. The parameter $\psi =(p1,p2,p3,p4,\lambda^{\text{ins}},\lambda^{\text{del}})$ is the parameter associated with sequencing error profile, which are defined in Table 1.

### 2.3 Full data likelihood

The full likelihood of observed reads ${\left\{R_{i}\right\}}_{i=1}^{n}$, reads membership ${\left[z_{ik}\right]}_{n\times K}$ and consensus ${\left\{C_{k}\right\}}_{k=1}^{K}$ is

(6) $$L=\prod_{i=1}^{n}\prod_{k=1}^{K}P(R_{i}|C_{k},\psi )P(z_{ik}=1|\alpha )P(C_{k}|r,\gamma_{k}),$$

where $\alpha =(\alpha_{1},\alpha_{2},\ldots ,\alpha_{K})$ with $\sum_{k=1}^{K}\alpha_{k}=1$ and the probability $P(z_{ik}=1)=\alpha_{k}$. The corresponding log-likelihood function is

(7) $$\begin{array}{c} l=\sum_{k=1}^{K}\sum_{i=1}^{n}z_{ik}[\text{log}(\alpha_{k})+n_{ik1}\;\text{log}(p_{1})+n_{ik2}\;\text{log}(p_{2})\\ +U_{ik}\;\text{log}(p_{3})+\sum_{u=1}^{U_{ik}}L_{iu}^{\text{del}}\;\text{log}(\lambda_{5})-\lambda^{\text{del}}U_{ik} \end{array}$$

 

$$\begin{array}{c} +W_{ik}\;\text{log}(1-p_{4})V_{ik}\;\text{log}(p_{4})+\sum_{v=1}^{V_{ik}}L_{iv}^{\mathrm{ins}}\;\text{log}(\lambda^{\text{ins}})-\lambda^{\text{ins}}V_{ik}]\\ +\sum_{j=1}^{3}n_{k,j}\;\text{log}(\gamma_{k,j})+N_{k}\;\text{log}(\beta_{k})+(L_{0}-N_{k})\;\text{log}(1-\beta_{k}). \end{array}$$

where $n_{ik1}=\sum_{l\in [s_{i},e_{i}]}1(Y_{\mathrm{ikl}}=1)$ and $n_{ik2}=\sum_{l\in [s_{i},e_{i}]}1(Y_{\mathrm{ikl}}=2)$.

We utilized the Classification Annealing Expectation Maximization algorithm (CAEM) for the inference, which contains the *AE*, *C*, and *M* steps. The details of the algorithm are provided in Section 1.2, available as supplementary data at *Bioinformatics Advances* online. The CAEM algorithm requires a predetermined number of consensus sequences, but the feature space is much larger than the number of samples in our model, making AIC and BIC inappropriate for the model selection (Giraud 2021). Thus, we adopt a five-fold cross-validation scheme to determine the optimal model based on the minimum error correction ratio score in the real application (Xie *et al.* 2016, Hashemi *et al.* 2018, Schrinner *et al.* 2020, Shaw and Yu 2022). As shown in Section 3.6, available as supplementary data at *Bioinformatics Advances* online, the MECR score is only slightly better when *K* is larger than the optimal model.

## 3 Simulation

To evaluate the performance of *DIHap*, we simulated the reads *via* the two-step generative process introduced in Section 2. For the number of haplotypes, we consider K∈{2,3} scenarios, representing the case of two normal consensus sequences, and two normal sequences with one disease-associated sequence, respectively. Typically, human cells have two consensus sequences, but certain cells, such as cancer cells, may develop subclones of chromosomes, thereby increasing the number of consensus sequences. Furthermore, some plants, fish, and yeast have more than two copies of their chromosomes, representing polyploid cases.

### 3.1 Simulation procedure

**Figure 2.**
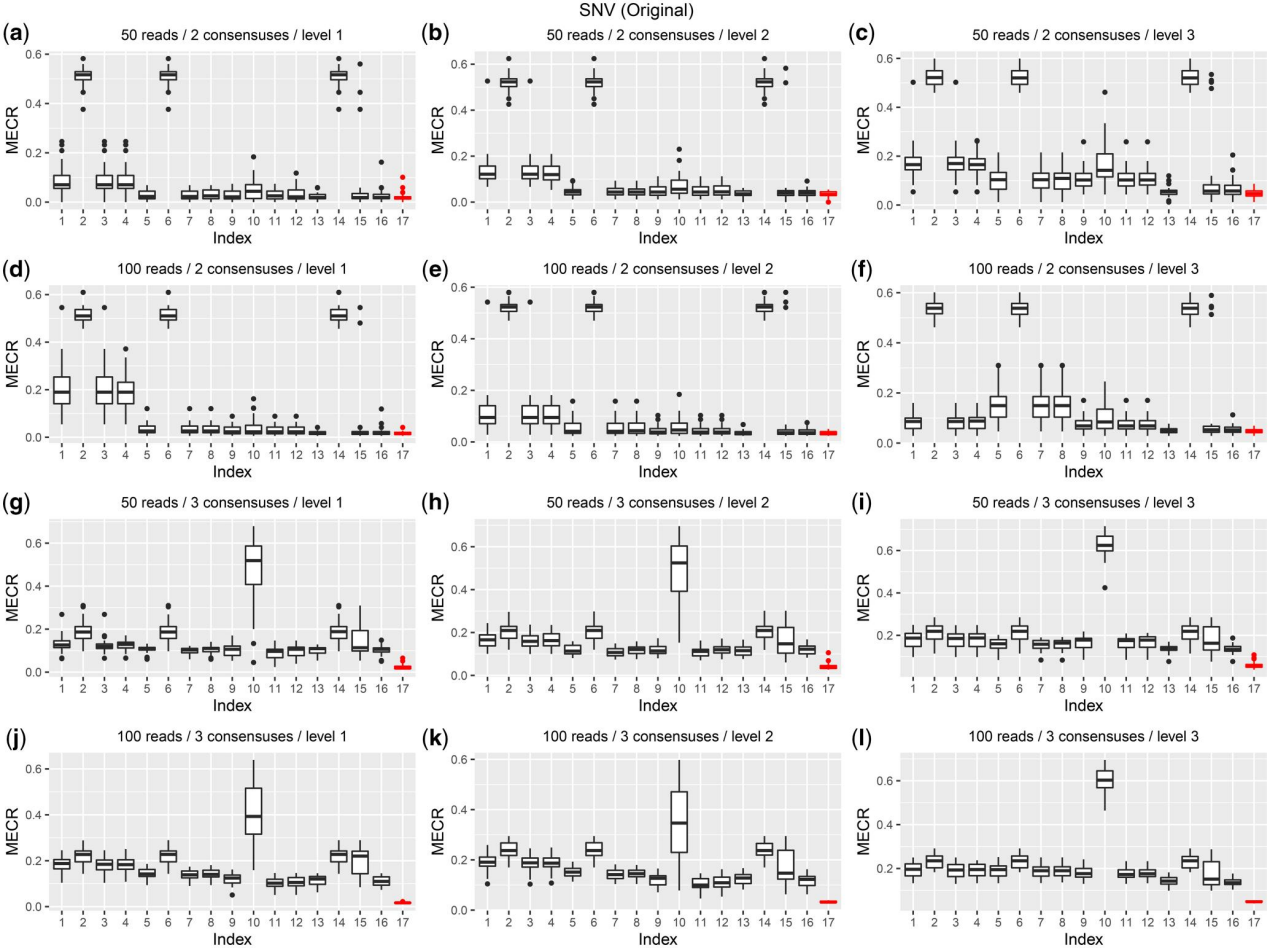
The MECR scores in Simulation 1. In this simulation, we have the true mapping status for each read. *DIHap* (17th bar) achieves the overall lowest MECR score, indicating consistent inference of the haplotype sequence and accurate classification of reads. The index numbers from 1 to 17 represent the combinations in Table 3.

**Figure 3.**
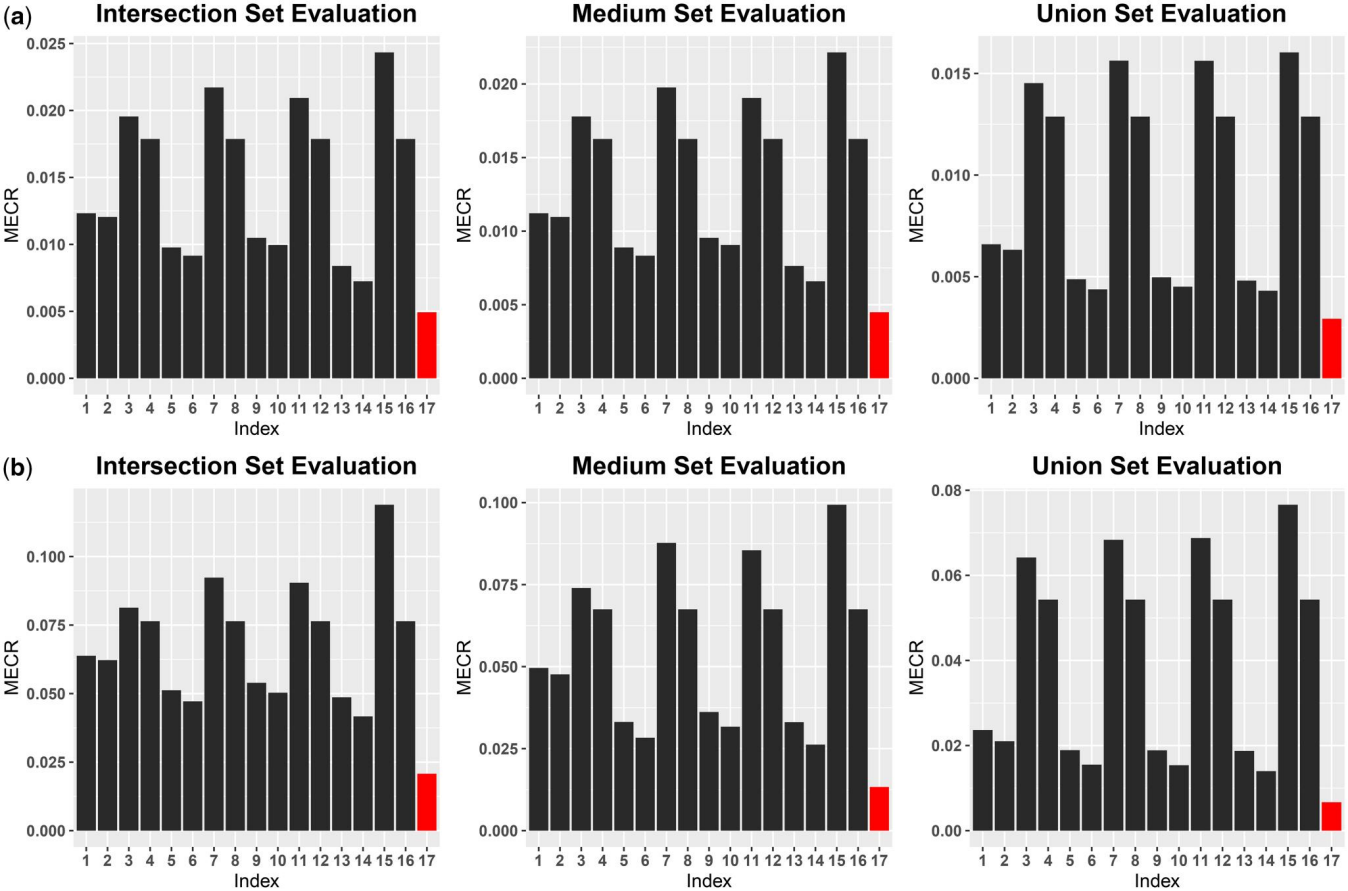
(a) The MECR scores achieved by *DIHap* and competing two-stage methods on the intersection, benchmark, and union sets for HG002 data. (b) The MECR scores achieved by *DIHap* and competing two-stage methods on the intersection, benchmark, and union sets for Otava data. *DIHap* (17th column) yields the smallest MECR scores on all three sets. The index numbers from 1 to 17 represent the combinations in Table 3.

The simulation of reads could be divided into two steps:

1. Generation of consensus sequences: Given the reference sequence, we first generate the consensus sequences with the evolution parameter γ. Suggested by (Erichsen and Chanock 2004, Chen *et al.* 2009, Zafar *et al.* 2016, The ICGC/TCGA Pan-Cancer Analysis of Whole Genomes Consortium 2020), we set $\gamma_{1}=\gamma_{2}=(0.9989,0.001,0.0001,0.0001)$ for K=2 and plus an additional sequence with higher mutation by $\gamma_{3}=(0.98572,0.0139,0.00038,0.00038)$ for K=3. The regions of the reference sequence utilized in the simulations are RECQL4 (chr8:144511288-144517833) and ERBB3 (chr16:56080108-56103505). In the simulations where structure variations (SVs) are introduced for consensus sequences (as shown in Section 2, available as supplementary data at *Bioinformatics Advances* online), five types of long variations are generated with the empirical probability shown in Fig. 3, available as supplementary data at *Bioinformatics Advances* online (MacDonald *et al.* 2014). The length of SVs is uniformly sampled from {50,100,500}.
2. Generation of reads: The proportion of read parameter is α=(0.6,0.4) for K=2 and α=(0.4,0.4,0.2) for K=3, respectively. The mapping lengths of reads are sampled from the empirical distribution (Fig. 2, available as supplementary data at *Bioinformatics Advances* online) of the real PacBio panel sequencing data. Then the starting position of the sequencing (or mapping) region is randomly selected from the targeted gene. Finally, as suggested in the previous literature (Weirather *et al.* 2017, Dohm *et al.* 2020), reads are generated with the error profile shown in Table 2. The error profiles of different TGS tools are shown in Table 4, available as supplementary data at *Bioinformatics Advances* online. The aforementioned parameter settings result in six-parameter setting scenarios.

**Table 2.** Error profiles in the simulations.^a^

Type of the sequencing error	Level 1 (%)	Level 2 (%)	Level 3 (%)
Mismatch	1.3000	1.4900	1.6800
Insertion	0.0870	4.0635	8.0400
Deletion	0.3400	1.7500	3.1600

a Level 1 and Level 3 are referred to the previous papers indicating the lower and higher sequencing error levels of the TGS. We set Level 2 as the midpoint between Level 1 and Level 3.

To assess the robustness of our algorithm, we conduct extensive simulations by varying several parameters, including the number of reads, other simulation software, alignment tools, and biased error profiles.

Simulation 1 is the full information case where the true mapping status of the reads is known. We set the number of reads n∈{50,100}, leading to 2×3×2=12 scenarios in the simulation 1. In simulation 2, we employ *minimap2* (Li 2018), one of the powerful mapping software tools, to map the reads to the reference sequence. It may introduce biases in the reads alignment procedure. In simulation 3, *PbSim2*, a tool developed to simulate the sequencing error profile of TGS, is employed to generate reads. It is a model misspecified simulation scenario, which can evaluate whether *DIHap* is robust across different reads simulation pipelines. Finally, to mimic the real sequencing data, SVs are introduced to the consensus sequences in simulation 4. The targeted gene in simulation 4 (ERBB3) differs from that in Settings 1 to 3 (RECQL4) to incorporate the SVs, while maintaining the same sequence depth as the previous conditions but with a larger number of reads.

### 3.2 Competing methods and evaluation metrics

To assess the performance of *DIHap*, we compare it with designed two-stage procedures. In the first stage, we utilize several popular variant detection methods, including *DeepVariant* (Poplin *et al.* 2018), *Clair3* (Zheng *et al.* 2022), *Bcftools* (Li *et al.* 2009), *FreeBayes* (Garrison and Marth 2012). In the second stage, the detected SNVs are phased by state-of-the-art phasing algorithms, such as *AltHap* (Hashemi *et al.* 2018), *flopp* (Shaw and Yu 2022), *HPOP* (Xie *et al.* 2016), and *WhatsHap* (Schrinner *et al.* 2020). As a result, 16 combinations of two-stage tools are obtained, as listed in Table 3. *DIHap* is the 17th method. In the simulated data where the ground truth of haplotypes is available, the correct phasing ratio (CPR) (Hashemi *et al.* 2018) between the inferred haplotypes $\hat{H}$ and the true haplotypes $H^{T}$ is the metric to evaluate the performance of haplotype inference, which is defined as

(8) $$\mathrm{CPR}(H^{T},\hat{H})=\frac{\sum_{k}\sum_{j\in D_{\chi (k)}}1({\hat{H}}_{k}(j)=H_{\chi (k)}^{T}(j))}{\sum_{k}|D_{\chi (k)}|},$$

where χ(k) is the one-to-one projection from the *k*th haplotype to the χ(k)th true haplotype and $D_{\chi (k)}$ is the set of the true SNVs in the χ(k)th true haplotype. The CPR score, falling into [0, 1], measures the dissimilarity between the inferred haplotypes and true haplotypes. Higher values indicate better results.

**Table 3.** Symbol of the combinations of the tools.^a^

Index	Combinations	Index	Combinations
1	*DeepVariant* × *AltHap*	2	*DeepVariant* × *flopp*
3	*DeepVariant* × *HPOP*	4	*DeepVariant* × *WhatsHap*
5	*Clair3* × *AltHap*	6	*Clair3* × *flopp*
7	*Clair3* × *HPOP*	8	*Clair3* × *WhatsHap*
9	*P-M-D* × *AltHap*	10	*P-M-D* × *flopp*
11	*P-M-D* × *HPOP*	12	*P-M-D* × *WhatsHap*
13	*FreeBayes* × *AltHap*	14	*FreeBayes* × *flopp*
15	*FreeBayes* × *HPOP*	16	*FreeBayes* × *WhatsHap*
17	*DIHap*		

a P-M-D represents the PEPPER-Margin-DeepVariant.

In the real application where true haplotypes are unavailable, we recommend using the minimum error correction ratio (MECR) score, which is defined as

(9) $$\mathrm{MECR}(R,\hat{H})=\frac{\sum_{i}\underset{k}{\min}\sum_{j\in O_{i}}1(R_{ij}\neq {\hat{H}}_{k}(j))}{\sum_{i}|O_{i}|},$$

where $O_{i}$ stands for the intersection of interest set of locations in the reference sequence with $R_{i}$. The MECR score, which ranges from 0 to 1, quantifies the probability of inconsistency per base between the reads R and inferred haplotypes $\hat{H}$. Compared to the traditional MEC criterion, the MECR score is normalized by the number of total bases of reads, ranging from 0 to 1 and providing a relative measure between the inferred haplotypes and the ground truth. To validate the effectiveness of MECR, we calculated the correlation between CPR and MECR in the simulated data. As shown in Section 3.3, available as supplementary data at *Bioinformatics Advances* online, MECR is negatively correlated with CPR, suggesting that the MECR score is an effective metric in situations where the true haplotypes are unavailable.

## 4 Results

In this section, we present a comprehensive comparison of haplotype-inference performance, benchmarking the methods on both simulated datasets and real-world sequencing data.

### 4.1 Simulation

Figure 2 illustrates the haplotype reconstruction performance of *DIHap* across the various scenarios of Simulation 1, comparing it with the competing two-stage methods in terms of MECR score. Overall, *DIHap* consistently achieves the smallest MECR score, indicating superior performance, especially for low coverage data (50 reads). Notably, in lower coverage and polyploid cases (50 reads and 3 consensuses), *DIHap* significantly outperformed competing two-stage alternatives. Figures 4–6, available as supplementary data at *Bioinformatics Advances* online, present MECR scores achieved by *DIHap* and competing methods from Simulations 2 to 4, detailed in Section 3.1, available as supplementary data at *Bioinformatics Advances* online. Consistent with the results from Simulation 1, *DIHap* demonstrates the best performance, indicating its robustness against different alignment tools and biased error profiles. It is noteworthy that *DIHap* demonstrates superior performance even under model misspecification with SVs in Simulation 4. The results of another evaluation metric, the CPR score, are shown in Figs 7–10, available as supplementary data at *Bioinformatics Advances* online, supporting the same conclusions as the MECR score.

If the haplotypes are obtained, it is obvious that we can determine whether a locus is a SNV or not. The byproduct of *DIHap* is SNV detection. The performance of *DIHap* is comparable to variant detection tools in the two haplotypes (or consensus sequences) cases. However, *DIHap* outperforms these tools in scenarios with three haplotypes, even under conditions of higher error rates and lower coverage. These findings indicate that *DIHap* is well-suited for haplotype inference in polyploid cases and population samples. A detailed comparison of the variant detection results can be found in Section 3.5, available as supplementary data at *Bioinformatics Advances* online.

### 4.2 Real data

In this section, we evaluated *DIHap* on a diploid TGS data HG002 (Zook *et al.* 2016) and a polyploid potato cultivar Otava TGS data (Sun *et al.* 2022), respectively.

#### 4.2.1 HG002

HG002 is a sample from a trio of Ashkenazim family cases, utilized as a benchmark by the Genome in a Bottle Consortium for evaluating variant detection and phasing tools. In our analysis, we employed PacBio sequencing files derived from the circular haplotype sequencing technique, which has an average read length of 15 kb and a coverage of 28. The reference genome used is the specific version hs37d5 of GRCh37 https://ftp-trace.ncbi.nlm.nih.gov/ReferenceSamples/giab/release/references/GRCh37/ and the mapping file can be downloaded from the ftp server https://ftp-trace.ncbi.nlm.nih.gov/giab/ftp/data/AshkenazimTrio/HG002_NA24385_son/PacBio_CCS_15kb/alignment/. We use the sequencing data from chromosome 22 as an illustrative example. Since the true SNV sites are unknown in real data, we designed three different SNV sets to evaluate the performance of *DIHap*. The first set is the intersection set of all methods, representing a scenario with a low false positive rate for variant detection. The second set, referred to as the medium set, is the union set of SNVs detected by at least three methods, which may correspond to a moderate false positive rate scenario. The third set is the union set of SNVs detected by all methods, representing a high false positive rate scenario. The true SNV set can be considered an intermediary between the union set and the intersection set.

The haplotype inference results of *DIHap* and the alternative two-stage methods presented in Table 3 are evaluated separately on the intersection, benchmark, and union sets. As illustrated in Fig. 3a, *DIHap* achieved the lowest MECR score compared to all combinations of detection and phasing algorithms across all three sets, resulting in more accurate haplotypes. Additionally, the performance of SNV detection can be assessed based on the benchmark set. As shown in Table 1, available as supplementary data at *Bioinformatics Advances* online, *DIHap* performs comparably to other algorithms. We utilized a Venn plot to visualize the overlap of the SNV detection results, as shown in Fig. 20, available as supplementary data at *Bioinformatics Advances* online.

#### 4.2.2 Potato cultivar otava

Potato is the most widely produced crop in the world, characterized by its four distinct haplotypes. However, reconstructing these haplotypes remains a challenging problem. The reference genome utilized in this study is DM1-3 516 R44 (downloaded from http://spuddb.uga.edu/pgsc_download.shtml). The corresponding PacBio HiFi sequencing files are available on the NCBI website, with the dataset number SRR15206231 (See the URL from https://www.ncbi.nlm.nih.gov/sra/SRR15206231). We employed *minimap2* with default parameters to map the sequencing files to the reference genome, focusing specifically on chromosome 2 of the potato for illustration.

Similar to HG002, we established the intersection set, benchmark set, and union set of SNVs to evaluate the performance of haplotype inference. As illustrated in Fig. 3b, *DIHap* significantly outperformed all two-stage procedures in the evaluation sets, demonstrating superior phasing capability in the polyploid context. In terms of detection performance, *DIHap* is comparable to *Clair3* but outperforms other methods, as shown in Table 3, available as supplementary data at *Bioinformatics Advances* online. The overlapped SNV detection results for all five methods are shown in Fig. 27, available as supplementary data at *Bioinformatics Advances* online.

Overall, *DIHap* demonstrates consistent inference capabilities on real data. The study of haplotype inference will enable researchers to gain insights into the correlations between variants, which is a crucial aspect of pharmacogenomics, providing multiple target regions for drug action. Additionally, investigating the haplotypes of potato can assist researchers in identifying key regions related to cultivation and pest resistance.

## 5 Conclusion

In this study, we propose a novel probabilistic framework, *DIHap*, designed for the direct estimation of haplotypes from sequencing data. This approach aims to reduce the accumulation of errors introduced by the conventional two-stage “detection-phasing” methodologies. *DIHap* models all loci instead of only variant loci, obtaining an accurate estimate for error profiles of sequencing data and increasing statistical power for low-coverage sequencing data. An efficient Classification Annealing Expectation Maximization algorithm has been designed to estimate haplotypes and model parameters. Extensive simulations have demonstrated that *DIHap* outperforms existing two-stage alternatives significantly in haplotype reconstruction tasks, particularly in handling challenging low-coverage and polyploid sequencing data scenarios. Furthermore, in the analysis of real diploid sequencing data such as HG002 and polyploid data from Potato Cultivar Otava, *DIHap* has exhibited superior performance in haplotype reconstruction compared to other two-stage methods. Besides, *DIHap* provides a general framework, capable of being adapted for different types of sequencing data, including both NGS and ONT data, even the integration of RNA-sequencing and DNA-sequencing data.

Several limitations of DIHap are worth investigating. First, DIHap does not consider long variations due to their low evolutionary probability and the difficulty of identifying breakpoints in low-coverage scenarios, possibly solvable by an MCMC sampling framework at the cost of high computational burden. Second, the assumption that haplotypes are evolved from the same reference sequence is not consistent with the biological process, where chromosome recombination can also lead to different haplotypes. Third, we would better cut the BAM file into several subregions for efficiency in real data applications (see Section 3.4 and 5, available as supplementary data at *Bioinformatics Advances* online).

## Supplementary Material

vbaf195_Supplementary_Data
