# Supplementary material for: Direct inference of haplotypes from sequencing data
Source: Bioinform Adv. 2025 Aug 20;5(1):vbaf195. doi: 10.1093/bioadv/vbaf195 (PMC12448230; doi:10.1093/bioadv/vbaf195)
Supplement: vbaf195_Supplementary_Data [file vbaf195_supplementary_data.pdf]

Supplemental Materials of "Direct Inference of Haplotypes from Sequencing Data"

Zhen Zhang[1,2,3,†], Bencong Zhu[3,†], Yongyi Luo[3], Jiandong Shi[3], Sheng Lian[3], Jingyu Hao[2], Taobo Hu[4], Toyotaka Ishibashi[5], Depeng Wang[6], Shu Wang *[4], Weichuan Yu †[2], and Xiaodan Fan ‡[3]

[1]The First Institute, Kunming Institute of Physics, Kunming, Yunnan Province, China

[2]Department of Electronic and Computer Engineering, The Hong Kong University of Science and Technology, Hong Kong SAR, China

[3]Department of Statistics, The Chinese University of Hong Kong, Hong Kong SAR, China

[4]Department of Breast Surgery, Peking University People's Hospital, Beijing, China

[5]Division of Life Science, Hong Kong University of Science and Technology, Hong Kong SAR, China

[6]GrandOmics Inc, Beijing, China

[†]These authors contributed equally to this work.

August 10, 2025

This file contains the supplemental materials of the paper titled "Direct Inference of Haplotypes from Sequencing Data". In Section S1, we provide the details of the CAEM algorithm. In Section S2, we extend our model to cover long insertion and deletion for the evolution step. Sections S3 and S4 contain additional results on simulations and two real sequencing datasets, respectively.

*Corresponding author Shu Wang: shuwang@pkuph.edu.cn

†Corresponding author Weichuan Yu: eeyu@ust.hk

‡Corresponding author Xiaodan Fan: xfan@cuhk.edu.hk

S1 Details of CAEM algorithm

S1.1 Data likelihood

Given sequencing error profile ψ, latent read membership $z_{ik} = 1$, and consensus sequence C_k, the observed data likelihood for each read R_i is

$$
\begin{aligned}
P(R_i|C_k,\psi) &= p_1^{n_{ik1}} p_2^{n_{ik2}} \times (p_3^{U_{ik}} \prod_{u=1}^{U_{ik}} p(L_{iu}^{del}|\lambda^{del})) \\
&\times (1-p_4)^{W_{ik}} (p_4^{V_{ik}} \prod_{v=1}^{V_{ik}} p(L_{iv}^{ins}|\lambda^{ins}).
\end{aligned}
$$

The definitions of values in the data likelihood are given by

$$
\begin{aligned}
n_{ik1} &= \sum_{l\in[s_i,e_i]} \mathbf{1}(R_i(l) = C_k(l)) = \sum_{l\in[s_i,e_i]} \mathbf{1}(Y_{ikl} = 1), \\
n_{ik2} &= \sum_{l\in[s_i,e_i]} \mathbf{1}(R_i(l) \neq C_k(l)) = \sum_{l\in[s_i,e_i]} \mathbf{1}(Y_{ikl} = 2), \\
W_{ik} &= e_i - s_i - 1 - 2\sum_{u=1}^{U_{ik}} L_{iu} - V_{ik} \\
U_{ik} &= \#\ deletion\ segements\ of\ R_i\ with\ C_k, \\
V_{ik} &= \#\ Insertion\ segements\ of\ R_i\ with\ C_k.
\end{aligned}
$$

$W_{ik}+V_{ik}$ represents all the gaps where insertion error can occur. The following probability density function models the lengths of insertion error and deletion error:

$$
\begin{aligned}
p(L_{iu}^{del}|\lambda^{del}) &= \text{Poi}(L_{iu}^{del} - 1|\lambda^{del}), \\
p(L_{iv}^{ins}|\lambda^{ins}) &= \text{Poi}(L_{iv}^{ins} - 1|\lambda^{ins}).
\end{aligned}
$$

The full likelihood of observed reads $\{R_i\}_{i=1}^n$, reads membership $[z_{i,k}]_{n\times K}$ and consensus $\{C_k\}_{k=1}^K$ is

$$
L = \prod_{i=1}^{n}\prod_{k=1}^{K} P(R_i|C_k,\psi)P(z_{i,k} = 1|\boldsymbol{\alpha})P(C_k|r,\boldsymbol{\gamma}_k),
$$

where $\boldsymbol{\alpha} = (\alpha_1, \alpha_2, \ldots, \alpha_K)$ with $\sum_{k=1}^K \alpha_k = 1$ and the probability $P(z_{ik} = 1) = \alpha_k$. The

corresponding log-likelihood function is

$$
\begin{aligned}
l &= \sum_{i=1}^{n}\sum_{k=1}^{K} z_{ik}(\log(\alpha_k) + n_{ik1}\log(p_1) + n_{ik2}\log(p_2) \\
&+ U_{ik}\log(p_3) + \sum_{u=1}^{U_{ik}} L_{iu}^{del}\log(\lambda^{del}) - U_{ik}(\log(\lambda^{del}) + \lambda^{del}) - \sum_{u=1}^{U_{ik}}\sum_{j=1}^{L_{iu}^{del}-1}\log(j) \\
&+ W_{ik}\log(1-p_4) + V_{ik}\log(p_4) + \sum_{v=1}^{V_{ik}} L_{iv}^{ins}\log(\lambda^{ins}) - V_{ik}(\log(\lambda^{ins}) + \lambda^{ins}) \\
&- \sum_{v=1}^{V_{ik}}\sum_{j=1}^{L_{iv}^{ins}-1}\log(j)) + \sum_{j=1}^{3} n_{kj}\log(\gamma_{kj}) + N_k\log(\beta_k) + (L_0 - N_k)\log(1-\beta_k)).
\end{aligned}
$$

S1.2 CAEM algorithm

We used the Classification Annealing Expectation Maximization algorithm (CAEM) to implement the inference [2]. CAEM comprise the AE, C, and M steps. In the AE step, the current probability of R_i belonging to the kth consensus in the mth step is

$$
t_{ik}^{(m)} = \frac{(\alpha_k P(R_i|C_k,\psi))^{1/\tau_m}}{\sum_{k'=1}^{K}(\alpha_{k'} P(R_i|C_{k'},\psi))^{1/\tau_m}},
$$

where, τ_m is the annealing parameter in the mth iteration. We update $\tau_{m+1} = \rho\tau_m$ with setting $\rho = 0.97$ as suggested [2].

In the C step, the label of R_i is randomly sampled from Categorical$(t_{i1}^{(m)}, \ldots, t_{iK}^{(m)})$. If the read is assigned to the kth consensus sequence, the $I_{ik}^{(m)} = 1$ otherwise 0.

In the M step, the Q function in the mth step is

$$
\begin{aligned}
Q(\boldsymbol{\alpha}, \boldsymbol{\gamma}, \psi, \mathcal{C}) &= \sum_{i=1}^{n}\sum_{k=1}^{K} I_{ik}^{(m)}(\log(\alpha_k) + n_{ik1}^{(m)}\log(p_1) + n_{ik2}^{(m)}\log(p_2) \\
&+ U_{ik}^{(m)}\log(p_3) + \sum_{u=1}^{U_{ik}^{(m)}}(L_{iu}^{(m)} - 1)\log(\lambda^{del}) - U_{ik}^{(m)}\lambda^{del} - \sum_{u=1}^{U_{ik}^{(m)}}\sum_{j=1}^{L_{iu}^{(m)}}\log(j) \\
&+ W_{ik}^{(m)}(\log(1-p_4)) + V_{ik}^{(m)}(\log(p_4)) \\
&+ \sum_{v=1}^{V_{ik}^{(m)}}(L_{iv}^{(m)} - 1)\log(\lambda^{ins}) - V_{ik}^{(m)}\lambda^{ins} - \sum_{v=1}^{V_{ik}^{(m)}}\sum_{j=1}^{L_{iv}^{(m)}-1}\log(j) \\
&+ \sum_{k=1}^{K}\sum_{j=1}^{3} n_{kj}^{(m)}\log(\gamma_{k,j}) + N_k^{(m)}\log(\beta_k) + (L_0 - N_k^{(m)})\log(1-\beta_k).
\end{aligned}
$$

Here, ${}^{s}n_{i,1}^{k(m)}, {}^{s}n_{i,2}^{k(m)}, W_i^{k(m)}, U_i^{k(m)}, V_i^{k(m)}$, and ${}^{e}n_{k,j}^{(m)}$ are the values in the mth iteration. In the M step, we update $\mathcal{C}$ and $\{\psi, \boldsymbol{\gamma}, \boldsymbol{\alpha}\}$ seperately.

After the S step, we know the group membership of each read. Without loss of generality, we take the kth group as an example. Firstly, we screen the distribution of the sites and classify the regions into two types: independent sites and indel blocks, as shown in Figure S1.

Figure S1 shows a toy example of the multiple sequence alignment result with the pseudo-reference sequence covering 16 bases. In this example, Base 1, 10, 11, and 14-16 are independent sites, because no reads contain either insertions or deletions compared to the reference sequence, while the remaining sites are in the indel block. For independent sites such as the lth location in the mth step, we perform the maximization step via

$$\begin{aligned} C_k^{(m)}(l) &= \arg\max_{b \in \mathcal{B}} \quad Q(\psi^{(m-1)}, \boldsymbol{\gamma}^{(m-1)}, \boldsymbol{\alpha}^{(m-1)}, C_k(l)) \\ &= \arg\max_{b \in \mathcal{B}} \sum_{i=1}^{n} I_{i,k}^{(m)} \log(P(R_i(l)|C_k(l) = b, \psi^{(m-1)})) \\ &+ \log(P(C_k(l) = b|r(l), \gamma_k^{(m-1)})), \end{aligned} \tag{S1}$$

where the base in the lth location of C_k is determined by the log-likelihood of read $R_i(l)$ and prior information $r(l)$. Due to independence, independent sites can be inferred in parallel.

For the sites in the indel block, we have to update them jointly. For example, to infer the jth indel block of C_k in the mth maximization step, which is denoted as $B_{k,j}^{(m)}$ ranging from $\mathcal{L} = [B_{k,j}^{(m)}(s)$ to $B_{k,j}^{(m)}(e)]$, we can sequentially update the block by

$$C_k^{(m)}(l) = \arg\max_{b \in \mathcal{B}} \sum_{i=1}^{n} I_{i,k}^{(m)} \log(P_1^{(m)}) + \log(P_2^{(m)}), \quad B_{k,j}^{(m)}(s) \le l \le B_{k,j}^{(m)}(e), \tag{S2}$$

where

$$\begin{aligned} P_1^{(m)} &= P\left(R_i|C_k^{(m)}(l'), C_k(l) = b, C_k^{(m-1)}(l''), \psi^{(m-1)}\right) \\ P_2^{(m)} &= P\left(C_k^{(m-1)}(l'), C_k(l) = b, C_k^{(m)}(l'')|r, \gamma_k^{(m-1)}\right) \end{aligned}$$

for $B_{k,j}^{(m)}(s) \le l' < l < l'' \le B_{k,j}^{(m)}(e)$.

After updating the C_k, other parameters in the mth iteration can be updated with the following

equations:

$$
\begin{aligned}
\alpha_k^{(m)} &= \frac{\sum_{i=1}^{n} I_{ik}^{(m)}}{n}, \quad k \in \{1, 2, ..., K\}, \\
p_1^{(m)} &= \frac{\sum_{i=1}^{n} \sum_{k=1}^{K} I_{ik}^{(m)} n_{ik1}^{(m)}}{\sum_{i=1}^{n} \sum_{k=1}^{K} I_{ik}^{(m)} (n_{ik1}^{(m)} + n_{ik2}^{(m)} + U_{ik}^{(m)})}, \\
p_2^{(m)} &= \frac{\sum_{i=1}^{n} \sum_{k=1}^{K} I_{ik}^{(m)} n_{ik2}^{(m)}}{\sum_{i=1}^{n} \sum_{k=1}^{K} I_{ik}^{(m)} (n_{ik1}^{(m)} + n_{ik2}^{(m)} + U_{ik}^{(m)})}, \\
p_3^{(m)} &= \frac{\sum_{i=1}^{n} \sum_{k=1}^{K} I_{ik}^{(m)} U_{ik}^{(m)}}{\sum_{i=1}^{n} \sum_{k=1}^{K} I_{ik}^{(m)} (n_{ik1}^{(m)} + n_{ik2}^{(m)} + U_{ik}^{(m)})}, \\
p_4^{(m)} &= \frac{\sum_{i=1}^{n} \sum_{k=1}^{K} I_{ik}^{(m)} V_{ik}^{(m)}}{\sum_{i=1}^{n} \sum_{k=1}^{K} I_{ik}^{(m)} (V_{ik}^{(m)} + W_{ik}^{(m)})}, \\
\lambda^{del(m)} &= \frac{\sum_{i=1}^{n} \sum_{k=1}^{K} I_{ik}^{(m)} \sum_{u=1}^{U_{ik}^{(m)}} L_{iu}^{(m)}}{\sum_{i=1}^{n} \sum_{k=1}^{K} I_{ik}^{(m)} U_{ik}^{(m)}} - 1, \\
\lambda^{ins(m)} &= \frac{\sum_{i=1}^{n} \sum_{k=1}^{K} I_{ik}^{(m)} \sum_{v=1}^{V_{ik}^{(m)}} L_{iv}^{(m)}}{\sum_{i=1}^{n} \sum_{k=1}^{K} I_{ik}^{(m)} V_{ik}^{(m)}} - 1.
\end{aligned}
$$

When the kth consensus sequences is compared to the reference sequence, the evolution parameter $\boldsymbol{\gamma}$ is updated via

$$
\begin{aligned}
\gamma_{kj}^{(m)} &= \frac{n_{kj}^{(m)}}{\sum_{j'=1}^{3} n_{kj'}^{(m)}}, \quad k \in \{1, 2, ..., K\}, \; j \in \{1, 2, 3\}, \\
\beta_k^{(m)} &= \frac{N_k^{(m)}}{L_0 - 1}, \quad k \in \{1, 2, ..., K\}
\end{aligned}
$$

Notice that, when the long variations are involved, we modify the evolution model with exponential-logarithmic distribution to describe the length patterns. The corresponding updating procedure is provided in Section S2. After the convergence of the CAEM algorithm, the corresponding haplotypes $\hat{\mathbf{H}}$ are obtained by extracting linked SNVs in consensus sequences $\hat{\mathbf{C}}$.

S2 Evolution Model with the Structural Variation (SV)

The evolution model in the main text assumes that the mutations include mismatch, insertion, and deletion of one site. The assumption is suitable for the case where the consensus sequences have no long variations, which is called structural variations (SV). In Simulation 4, we introduce five types of SV with probability based on the empirical distribution of data in the Genomic Variants Database (DGV) [3](shown in Figure S3) for each consensus sequence.

To account for SVs, we modify the evolution part in *DIHap* by considering the length of insertion and deletion segments. The SVs, duplication and translocation, can be viewed as special combinations of the long deletion and insertion. The inversion is composed of continuous small

indels and SNVs. Hence, the SV blocks are tackled by modeling the length of long deletion and insertion segments. As suggested by [1], we assume the lengths of long deletion (L^{DEL}) and insertion (L^{INS}) mutations follow the exponential-logarithms distribution:

$$
\begin{aligned}
p(L^{DEL}|h_5,\beta_5) &= \frac{-1}{\log(h_5)}\frac{\beta_5(1-h_5)e^{-\beta_5 L^{DEL}}}{1-(1-h_5)e^{-\beta_5 L^{DEL}}},\\
p(L^{INS}|h_6,\beta_6) &= \frac{-1}{\log(h_6)}\frac{\beta_6(1-h_6)e^{-\beta_6 L^{INS}}}{1-(1-h_6)e^{-\beta_6 L^{INS}}},
\end{aligned}
$$

where (h_5,β_5) and (h_6,β_6) are parameters of the density function for deletion and insertion length, respectively.

For the inference, the modification has no influence on the sequencing error parameter estimation. We only have to estimate the parameters (h_5,β_5) by

$$
\beta_5^{(m+1)} = n_k^{(m)}\left(\sum_{j=1}^{n_k^{(m)}}\frac{L_j^{DEL}}{1-(1-h^{(m)})e^{-\beta_5^{(m)}L_j^{DEL}}}\right)^{-1},
$$

$$
h^{(m+1)} = \frac{-n_k^{(m)}(1-h^{(m)})}{ln(h^{(m)})\sum_{j=1}^{n_k^{(m)}}(1-(1-h^{(m)})e^{-\beta_5^{(m)}L_j^{DEL}})^{-1}},
$$

where $n_k^{(m)}$ denotes the number of deletion mutations in the evolution stage for the kth consensus sequence. L_j^{DEL} is the length of the jth deletion mutation for the kth consensus sequence. Similar results can be obtained for (h_6,β_6). In Simulation 4 and real application of the main text, we used the modified model considering the length of insertion and deletion segments.

S3 Additional Simulation Results

S3.1 MECR scores of Simulation 2 to 4

In the main content, we present the phasing results of Simulation 1 based on the MECR score. In this subsection, we provide results from Simulations 2 to 4. In summary, *DIHap* is comparable to other competing methods in the case of $K = 2$ haplotypes (consensus sequences) and outperforms them in the $K = 3$ scenario under biased mapping reads (Simulation 2), varying error profile assumptions (Simulation 3), and structural variations (SVs) (Simulation 4). The results are illustrated in Figures S4 to S6.

S3.2 CPR Scores of Simulation 1 to 4

In the subsection, we provide the CPR scores for the 16 two-stage tools and *DIHap* (the 17th bar in the bar plot) from Figures S7 to S10, which represent the outcomes from Simulations 1 to 4. The conclusions drawn from the CPR scores are consistent with those from the MECR scores. *DIHap* demonstrates robustness to mapping errors and SVs, achieving superior performance in haplotype inference, particularly in polyploid cases.

S3.3 Relation between CPR score and MECR score

When the ground truth of haplotypes is available, the performance of haplotype inference can be assessed using the CPR score, which quantifies the difference between the true haplotypes and the estimated haplotypes. A higher CPR score indicates a smaller discrepancy between the inferred and true haplotypes. In the absence of true haplotypes, the MECR score is utilized. To validate the effectiveness of MECR, we calculated the correlation between CPR and MECR using simulated data. The results, presented in Figures S11 to S14, demonstrate a negative correlation between CPR and MECR. Thus, MECR serves as an effective metric for evaluating haplotype inference when the true haplotypes are unavailable.

S3.4 Switch error rate of Simulation 1 to 4

MECR and CPR evaluate the performance of haplotype inference based on the hamming error in a local scale. To evaluate the consecutive consistence of SNVs globally, switch error rate (SER) is adopted to perform the comparison. SER is defined as Eq.(S3):

$$SER = \frac{SP}{AP}, \tag{S3}$$

where SP is the number of neighboured pairs of SNVs and AP is the all the pairs of neighboured SNVs. In Figure S15 (a), there are 7 SNVs (6 effective pairs, $AP = 6$) and 1 switch between 3rd and 4th SNV site ($SP = 1$). Therefore, SWR for case (a) is $\frac{1}{6}$. In Figure S15 (b), there are also 7 SNVs but with wrong inference 4th SNV, which excludes the pair of 3rd and 4th SNV, and 4th and 5th SNV ($AP = 4$). Also one switch occurs between 4th and 5th SNV ($SP = 1$). As a result, SER for case (b) is $\frac{1}{4}$.

With SER, we compare the inference results of all the combinations of the methods with *DIHap*, displayed from Figure S16 to Figure S19. As aforementioned case (b) in Figure S15, the comparison results indicate the the consistent result with CPR results, and *DIHap* has relative small error rate and higher CPR score especially in the polyploid case.

S3.5 SNV detection performance

The byproduct of *DIHap* is the SNV identification. We evaluate the performance of SNV identification on simulated data for *DIHap* and competing variant detection methods. Suppose that we have detected a SNVs (denoted as A set), and there are b true SNVs (denoted as B set). A detected SNV is considered a match to the truth if and only if both the variant content and its location equal precisely to those in the true dataset. If c true SNVs are identified, then the

evaluation metrics, precision, recall, F1 score, FM, and Jaccard, are defined by

$$\begin{aligned} precision &= \frac{c}{a} \\ recall &= \frac{c}{b} \\ F1\ score &= \frac{precision + recall}{2} \\ FM &= \sqrt{precision \times recall} \\ Jaccard &= \frac{c}{a+b-c}. \end{aligned}$$

This paragraph provides the results of SNV detection from Simulation 1 to Simulation 4. As shown in Figure S20, the performance of *DIHap* and other competing variant calling algorithms is comparable under diploidy scenarios of Simulation 1, but *DIHap* performs better under polyploid scenarios. In Simulation 2 (shown in Figure S21), we use the *minimap2* to map the reads with respect to the reference sequence. Compared to Simulation 1, the mapping procedure introduces mapping errors, leading to worse SNV identification results for all methods. In this simulation, *DIHap* also achieved the best performance, especially for the polyploid case. Simulation 3 (shown in Figure S22) replaces the read generation procedure with the scheme provided by *Pbsim2*. Under the misspecified simulation mechanisms, *DIHap* also outperforms others in all cases, illustrating the robustness of *DIHap*. To evaluate the performance of *DIHap* under the existence of SVs, sequencing data with SVs are generated in Simulation 4. Figure S23 presents the results in Simulation 4. Compared to the previous simulations, the performances of all methods have decreased. However, *DIHap* still outperforms other competing methods in the polyploid case. These simulations illustrate that *DIHap* is robust across different simulation scenarios.

S3.6 Selection of K in Sequencing Data

In haplotype inference algorithms, the number of haplotypes (or consensus sequences), denoted as K, is often unknown in real data due to cell impurity or polyploidy. We recommend using a five-fold cross-validation procedure for selecting K based on the MECR score. A simulation study was conducted to demonstrate the performance of this procedure. In Figure S24, we followed the simulation protocol with three haplotypes and 100 reads. Under the five-fold design, 80 reads were selected as the training sample, while the remaining reads served as validation samples. The MECR score was calculated on the training samples, and this procedure was replicated five times. As shown in Figure S24, when $K < 3$, the MECR score drops sharply across all replications, while the score stabilizes when $K = 3$, indicating that $K = 3$ is the elbow point. This behavior can be explained by the fact that when K matches the true number of haplotypes, the algorithm can accurately recover most haplotypes with a small MECR score. Conversely, if K exceeds the true number, at least two haplotypes will share most of the content, leading to overfitting. Therefore, the elbow point identified through five-fold cross-validation serves as a reliable model selection scheme.

We also demonstrate another simulation case with 4 haplotypes in truth. In figure S25, we also followed the simulation protocol with three haplotypes and 200 reads. Under the five-fold

design, 160 reads were selected as the training sample, while the remaining reads served as validation samples. The MECR score was calculated on the training samples, and this procedure was replicated five times. Simlilarly, $K = 4$ hits the elbow point.

S4 Additional Real Application Results

S4.1 Instructions of Reprocessing of DIHap

Before implementation of *DIHap*, the data needs the following prepossessing steps:

i Collect the sequencing data. The format of the data is FASTA or FASTQ. The detail information of these formats can be checked from https://compgenomr.github.io/book/fasta-and-fastq-formats.html.

ii Mapping the sequencing data with the default parameters of mapping software. For the third-generation sequencing data, we recommend to use *Minimap2*. The instructions can be retrieved from the webpage https://github.com/lh3/minimap2. However, for public data like HG002, we recommend to use the mapped reads in BAM or SAM form (note for the mapping file: https://zymoresearch.eu/blogs/blog/what-are-sam-and-bam-files?srsltid=AfmBOoqvwzyQGJYEFqkr9iVMVwi5iWHcRMViBjxsyKZcOSgeSmo_TmMn) to save time.

iii (Option pipline) If the target bam span a very long range, we recommand to split the bam into different subregions.

- Scan the bam to find the boundary of the insertion and deletion per read. We take the forbidden union of the boundary sites.
- Determine the length and form the initial range of the subregion. (for example 200Mb for HG002 data.)
- We expand the intial range until both boundaries of subregion overlapped with the forbidden union.
- Cut the bam based on the updated range.

After these prepossessing procedures, readers can follow the website to install and use *DIHap* (https://github.com/new-zbc/DIHap).

S4.2 SNV detection performance

In this section, we provide the result of SNV detection in the real sequencing data. Since we do not have the ground truth of SNVs in the real data, we construct the benchmark set by using the overlapped SNVs detected by different tools. The benchmark set includes the variants detected by at least 3 methods, as shown in Figure S26. Based on the benchmark set, the performance of *DIHap* and competing methods is evaluated. The results in Table S2 implies that the performances of *DIHap* and other baseline tools are comparable.

Similarly, the benchmark set is also constructed based on the SNV detection results for Otava data (Figure S27). Based on the benchmark set, the performance of variant detection is shown in Table S3, where all methods performs similarly.

S5 Running Time

We have included the time cost of DIHap based on 43 regions of HG002, as shown in Figure S29. The average computation time is 22,781.29 seconds, with a standard deviation of 25,656.67 seconds.

The time consumption is influenced by both the length of the subregions and the coverage of the reads. Since the length of the subregions is consistently set at 809,815 bases, we present the coverage of the different subregions in Figure S29. With the exception of Subregion 5, the coverage across the other subregions is approximately 30.

Additionally, we calculated the computation time per base for the subregions, as illustrated in Figure S30. Although Subregion 5 has lower coverage compared to the other subregions, its initialization step shows a slightly higher running time. Overall, the running time per base and per haplotype is 0.0007 seconds, with a standard deviation of 0.0017 seconds.

References

[1] D. Beyter, H. Ingimundardottir, A. Oddsson, H. P. Eggertsson, E. Bjornsson, H. Jonsson, B. A. Atlason, S. Kristmundsdottir, S. Mehringer, M. T. Hardarson, et al. Long-read sequencing of 3,622 Icelanders provides insight into the role of structural variants in human diseases and other traits. *Nature Genetics*, 53(6):779–786, 2021.

[2] G. Celeux and G. Govaert. A classification EM algorithm for clustering and two stochastic versions. *Comput. Stat. Data Anal.*, 14(3):315–332, 1992.

[3] J. R. MacDonald, R. Ziman, R. K. Yuen, L. Feuk, and S. W. Scherer. The database of genomic variants: a curated collection of structural variation in the human genome. *Nucleic Acids Research*, 42(D1):D986–D992, 2014.

Table S1: The performance of the detection on HG002 chromosome 22 based on the medium SNV set
. *DIHap* shows a comparable detection performance to other methods based on F1 score, FM and Jaccard.

Algorithms	Precision	Recall	F1 score	FM	Jaccard
DeepVariant	0.9962	0.8677	0.9275	0.9297	0.8648
Clair3	0.9833	0.9859	0.9846	0.9846	0.9697
PEPPER-Margin-DeepVariant	0.9915	0.9702	0.9807	0.9808	0.9622
FreeBayes	0.8978	0.9923	0.9427	0.9438	0.8912
DIHap	0.8929	0.9692	0.9295	0.9303	0.8683

Table S2: The performance of the detection on HG002 chromosome 22 based on the benchmark SNV set. *DIHap* shows a comparable detection performance to other methods based on F1 score, FM and Jaccard.

Algorithms	Precision	Recall	F1 score	FM	Jaccard
DeepVariant	0.8322	0.9584	0.8909	0.8931	0.8033
Clair3	0.7517	0.9966	0.8570	0.8656	0.7498
PEPPER-Margin-DeepVariant	0.7707	0.9971	0.8694	0.8766	0.7690
FreeBayes	0.6823	0.9972	0.8102	0.8249	0.6810
DIHap	0.6814	0.9779	0.8031	0.8163	0.6711

Table S3: The performance of the detection on Otava chromosome 2 based on the medium SNV set. *DIHap* shows a comparable detection performance to *Clair3* and *PEPPER-Margin-DeepVariant*, outperorming others based on F1 score, FM and Jaccard.

Algorithms	Precision	Recall	F1 score	FM	Jaccard
DeepVariant	0.9885	0.5178	0.6796	0.7154	0.5147
Clair3	0.9593	0.9258	0.9422	0.9424	0.8907
PEPPER-Margin-DeepVariant	0.9629	0.8737	0.9161	0.9172	0.8453
FreeBayes	0.5984	0.9358	0.7300	0.7483	0.5748
DIHap	0.7305	0.9502	0.8260	0.8331	0.7036

Table S4: Error level of some TGS tools. The sequencing error varies among the sequencing technologies and versions of the tools.

Type of Error	PacBio	PacBio (CCS)	ONT
Mismatch (%)	1.68	0.34	4.33
Insertion (%)	8.04	0.87	3.69
Deletion (%)	3.16	1.3	4.54

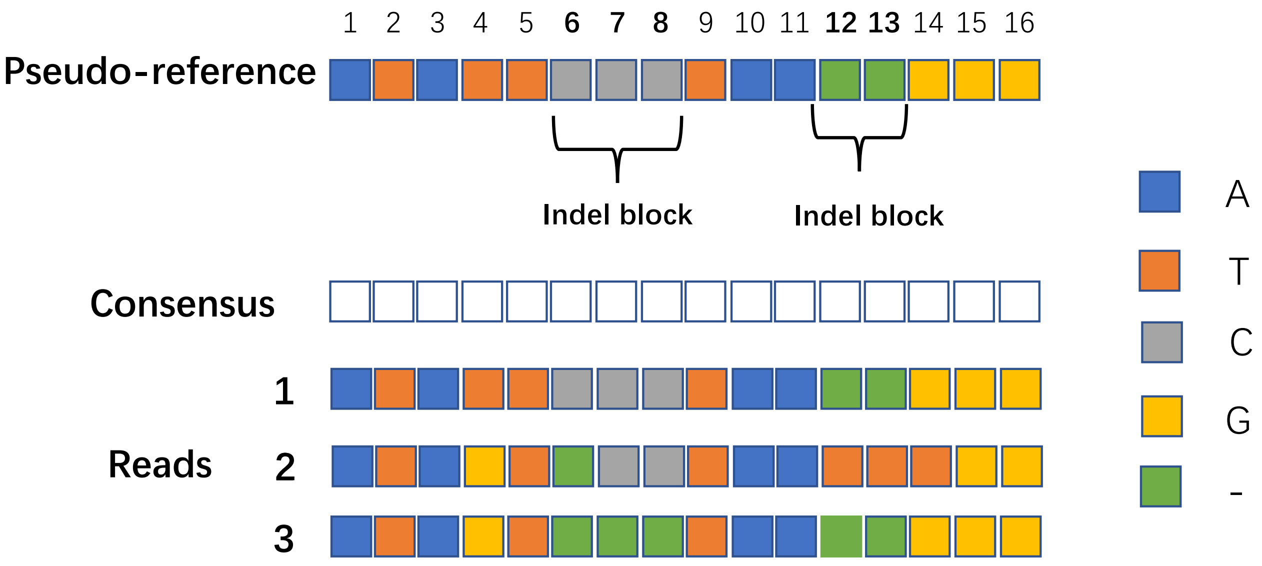

Figure S1: One example for inference algorithm. Psedo-reference is constructed by the multiple sequence alignment result of the reads, where green box (i.e. " $-$ ") of Base 12 and 13 in the pseudo-reference are introduced by the Read 2 with two more "T" nucleoside between Base 11 and 14. Thus, Base 12 and 13 are grouped as one indel block. Similarly, Base 6 of the Read 2 and Base 6-8 of the Read 3 delete 1 and 3 "C" nucleoside respectively, forming one indel block. Other sites except for ones within indel blocks are regarded as independent sites. Given the pseudo-reference and reads, indel blocks are separated for consensus sequence inference with Eq.(S2) and remaining sites are updated with Eq.(S1).

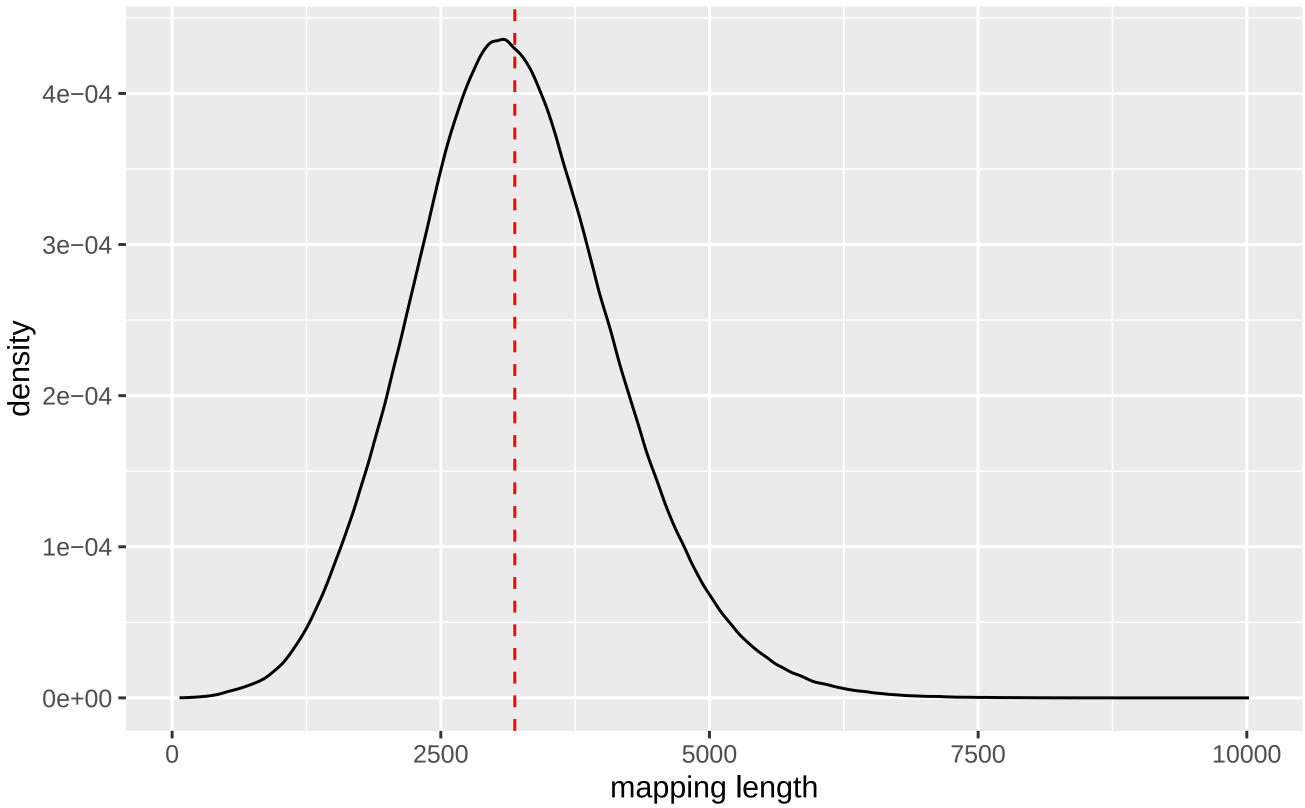

Figure S2: Empirical length of the reads in simulations. The dashed line denotes the average length, around 3000 bases.

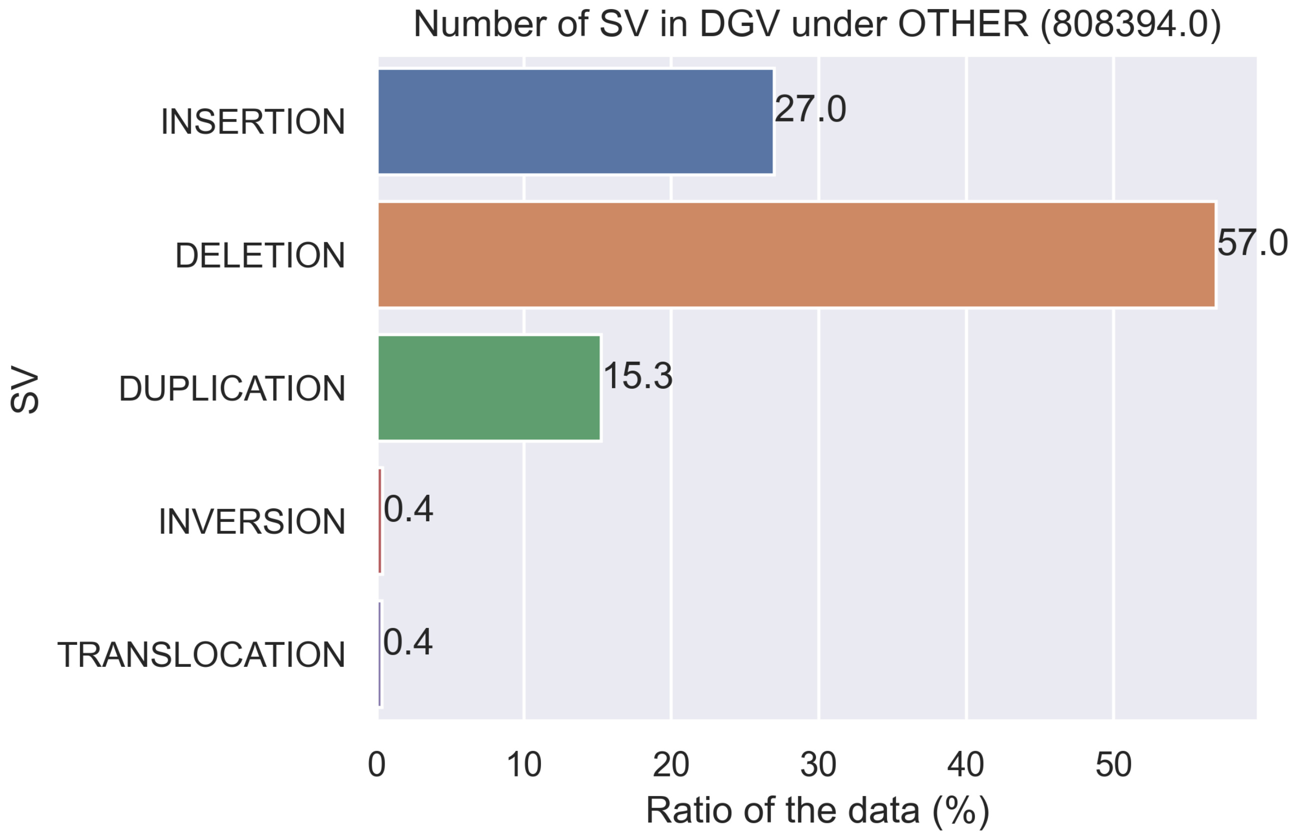

Figure S3: Portions of the long variations in DGV.

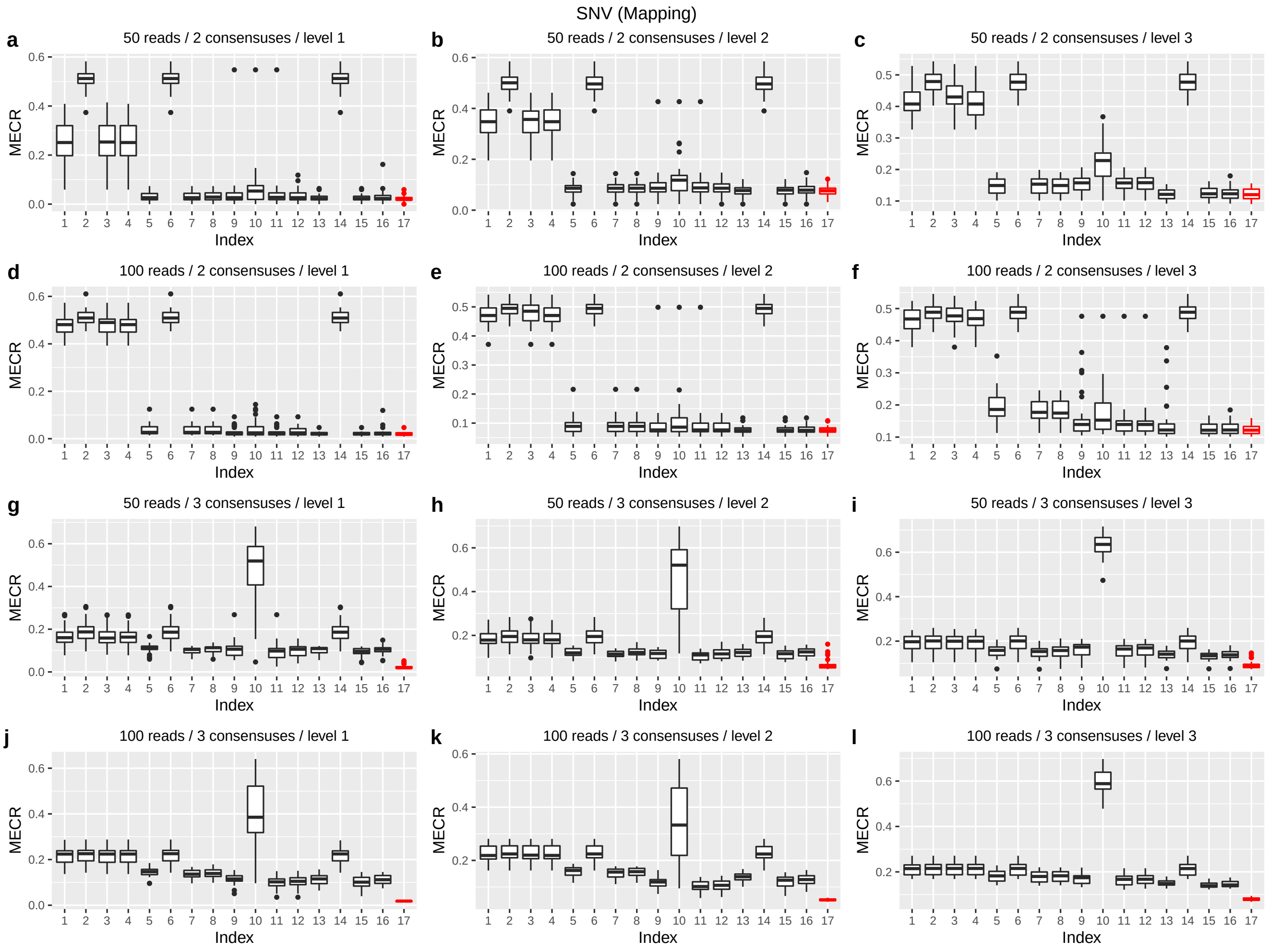

Figure S4: The MECR score of haplotype inference in Simulation 2. Simulation 2 builds upon Simulation 1 by further introducing alignment bias into the results. Since *DeepVariant* failed to detect SNVs at error levels 2 and 3, its performance in haplotype inference was adversely affected (as shown in Bars 1 to 4). In contrast, *DIHap* demonstrates the advantages of an integrated approach, achieving a lower MECR score, particularly in the case of three haplotypes.

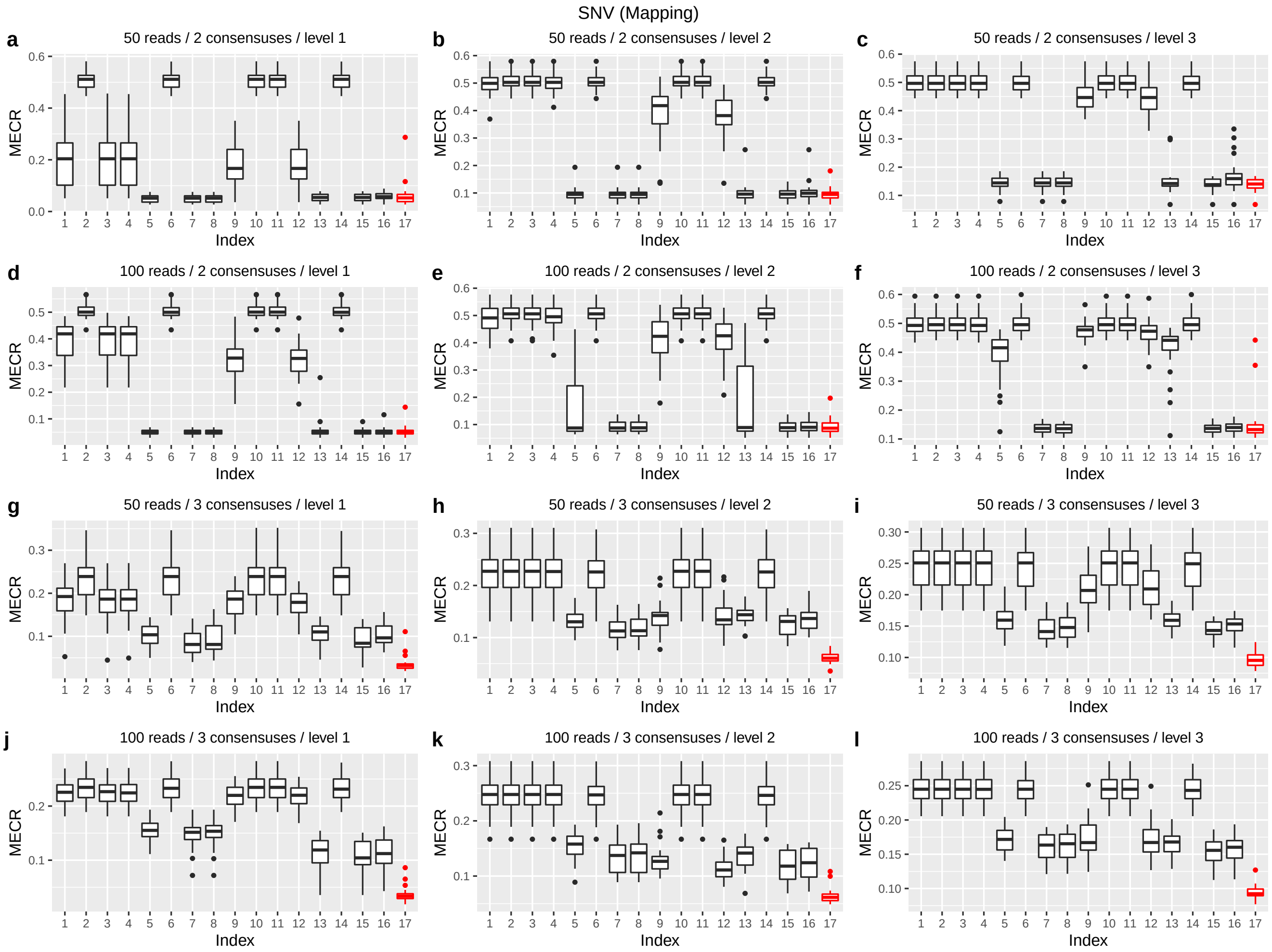

Figure S5: The MECR score of haplotype inference in Simulation 3. Simulation 3 further employs *PbSim2* to simulate reads based on the haplotype sequences. *DIHap* demonstrates robustness to the sequencing error profile generated by *PbSim2*, achieving a lower MECR score even under conditions of lower coverage and in the case of three haplotypes.

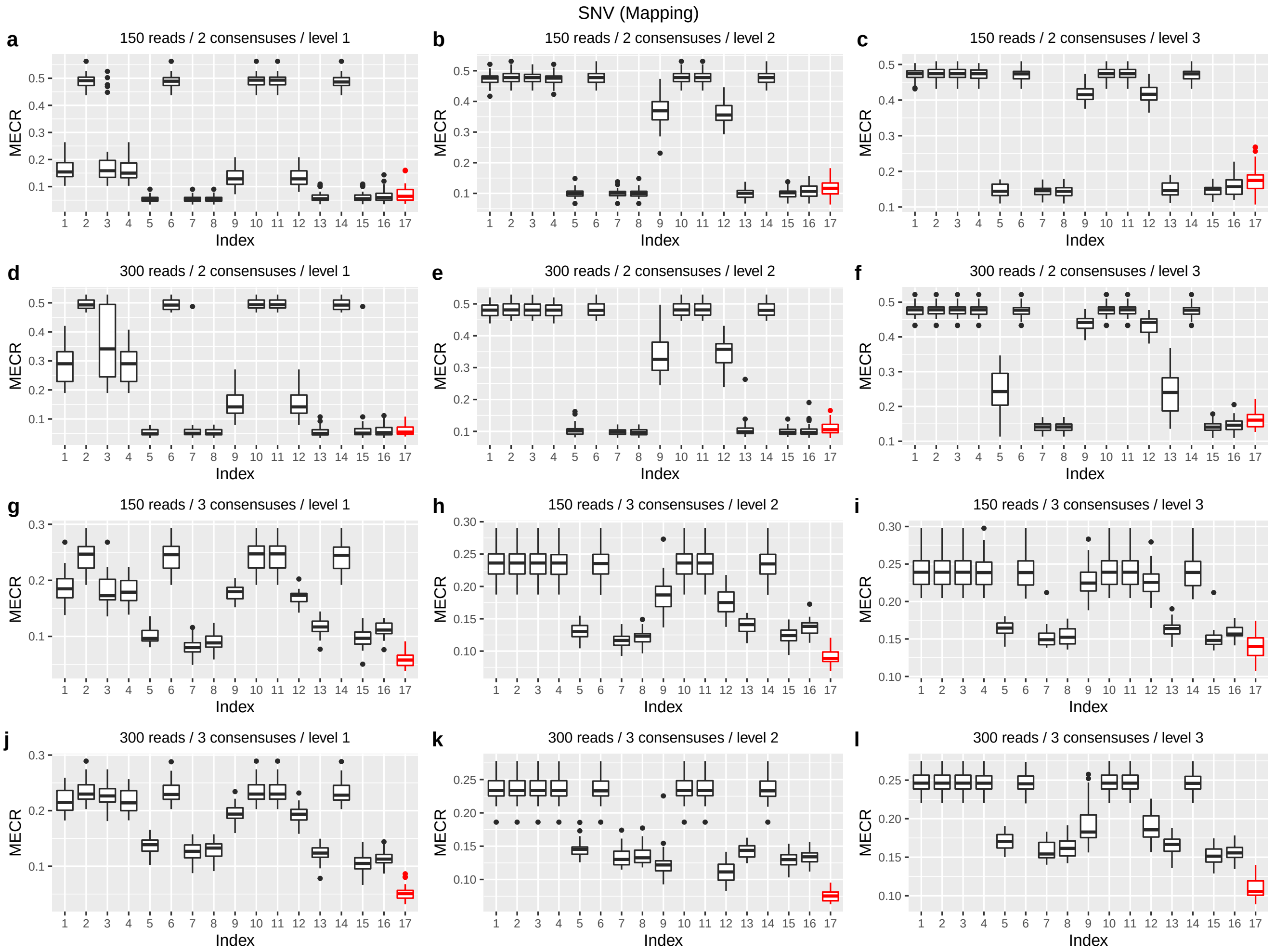

Figure S6: The MECR score of haplotype inference in Simulation 4. Simulation 4 introduces the structural variations to the consensus sequences. In this simulation, *DIHap* also yields a comparable MECR score in the 2 haplotypes (or consensus sequences) case and performs best in 3 haplotypes case.

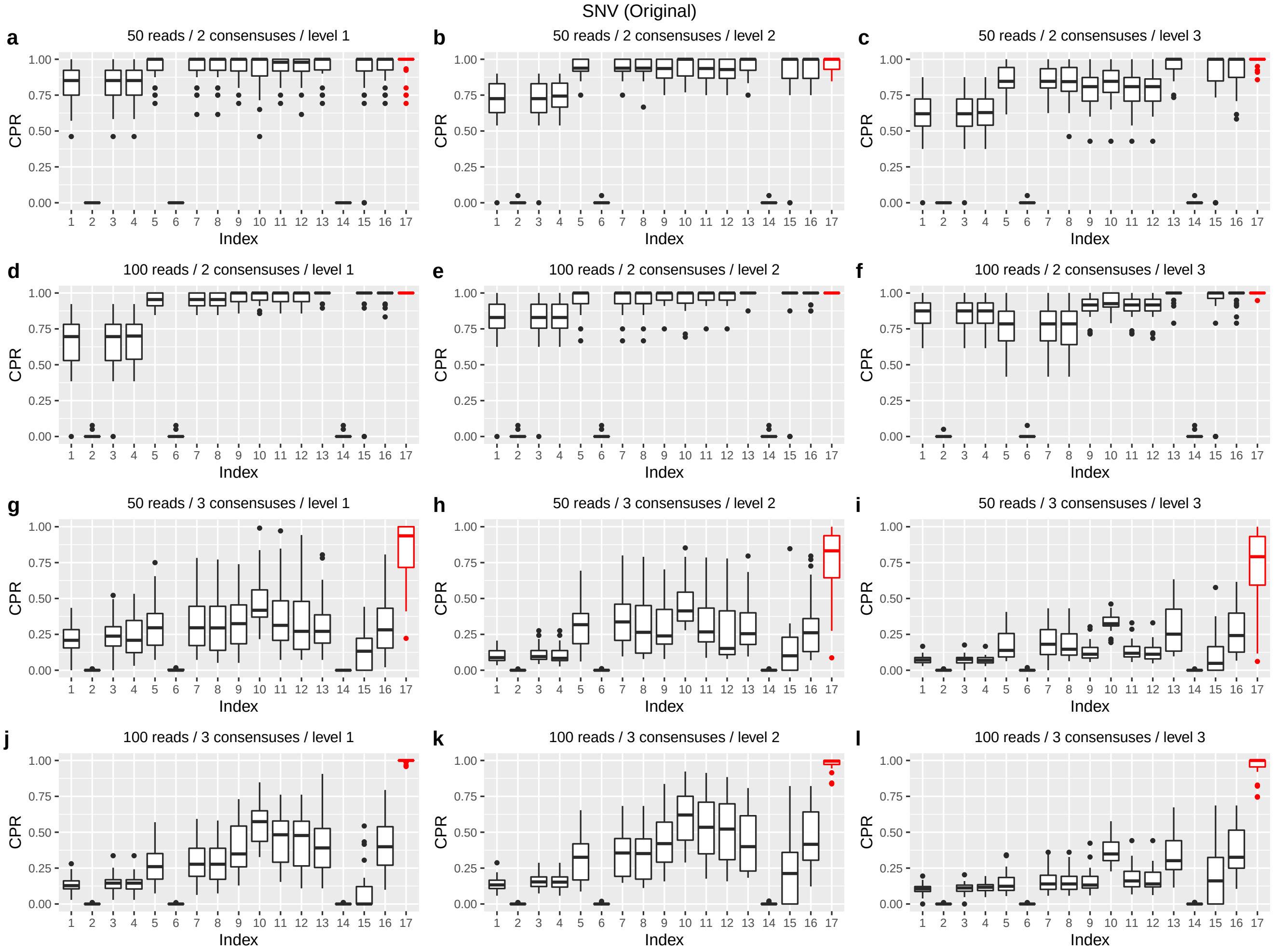

Figure S7: CPR score of the Simulation 1. In this case, we have the true mapping status of each read. And *DIHap* (17th bar) can achieve overall highest CPR score, indicating the consistent inference of the haplotypes.

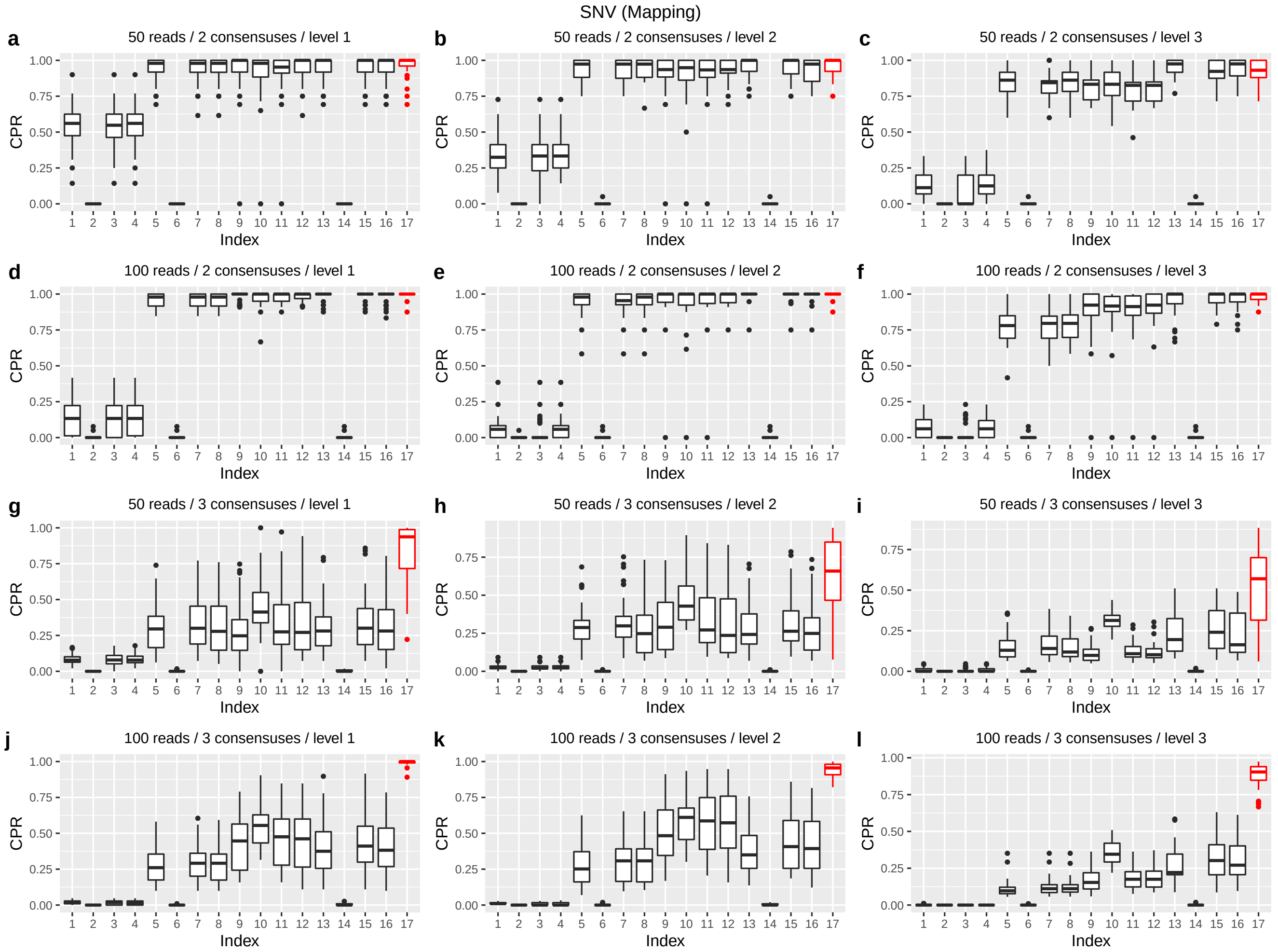

Figure S8: CPR score of the Simulation 2. Simulation 2 is based on Simulation 1, further introducing the alignment bias to the result. *DIHap* also shows the comparable CPR score to the combinations of the tools and outperforms them for 3 consensus sequences (or haplotypes) case.

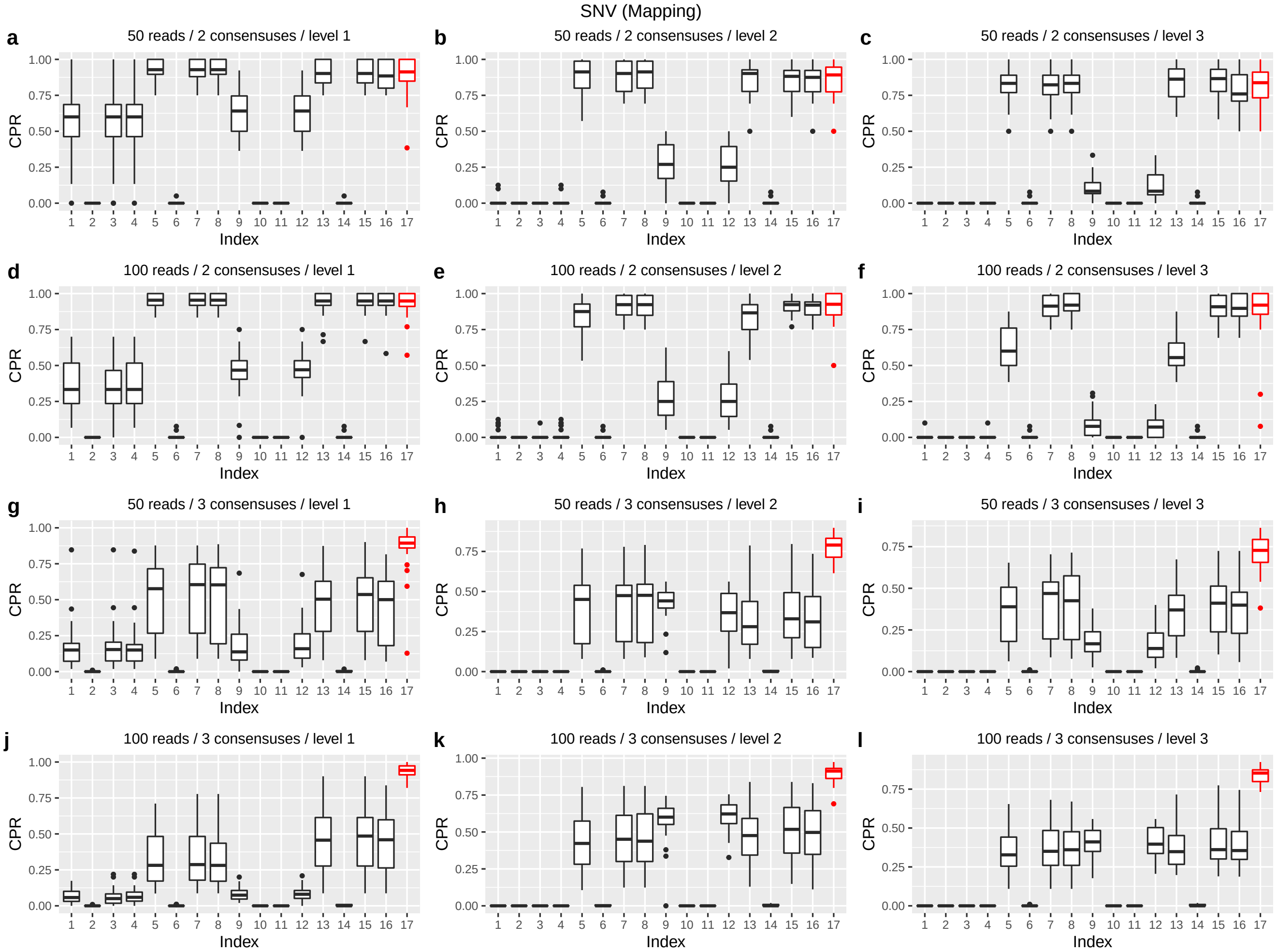

Figure S9: CPR score of the Simulation 3. Simulation 3 further adopts *PbSim2* to simulate the reads given the consensus sequences. *DIHap* shows the robustness against the sequencing error profile.

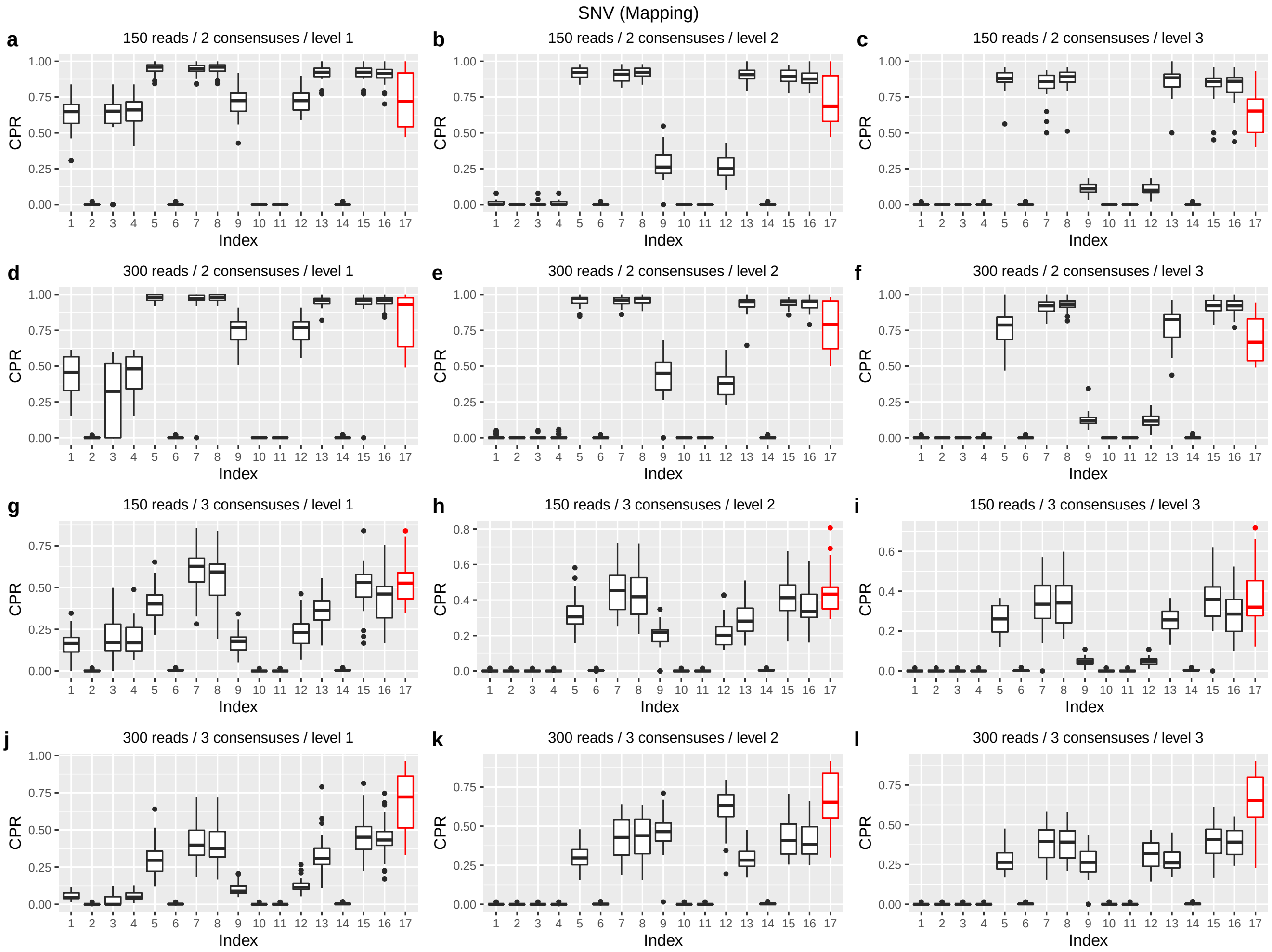

Figure S10: CPR score of the Simulation 4. Simulation 4 introduces the structural variations to the consensus sequence. *DIHap* also achieves a comparable CPR score of the inference in the 2 consensus sequences case and outperforms others in 3 consensus sequences (or haplotypes) case with higher coverage.

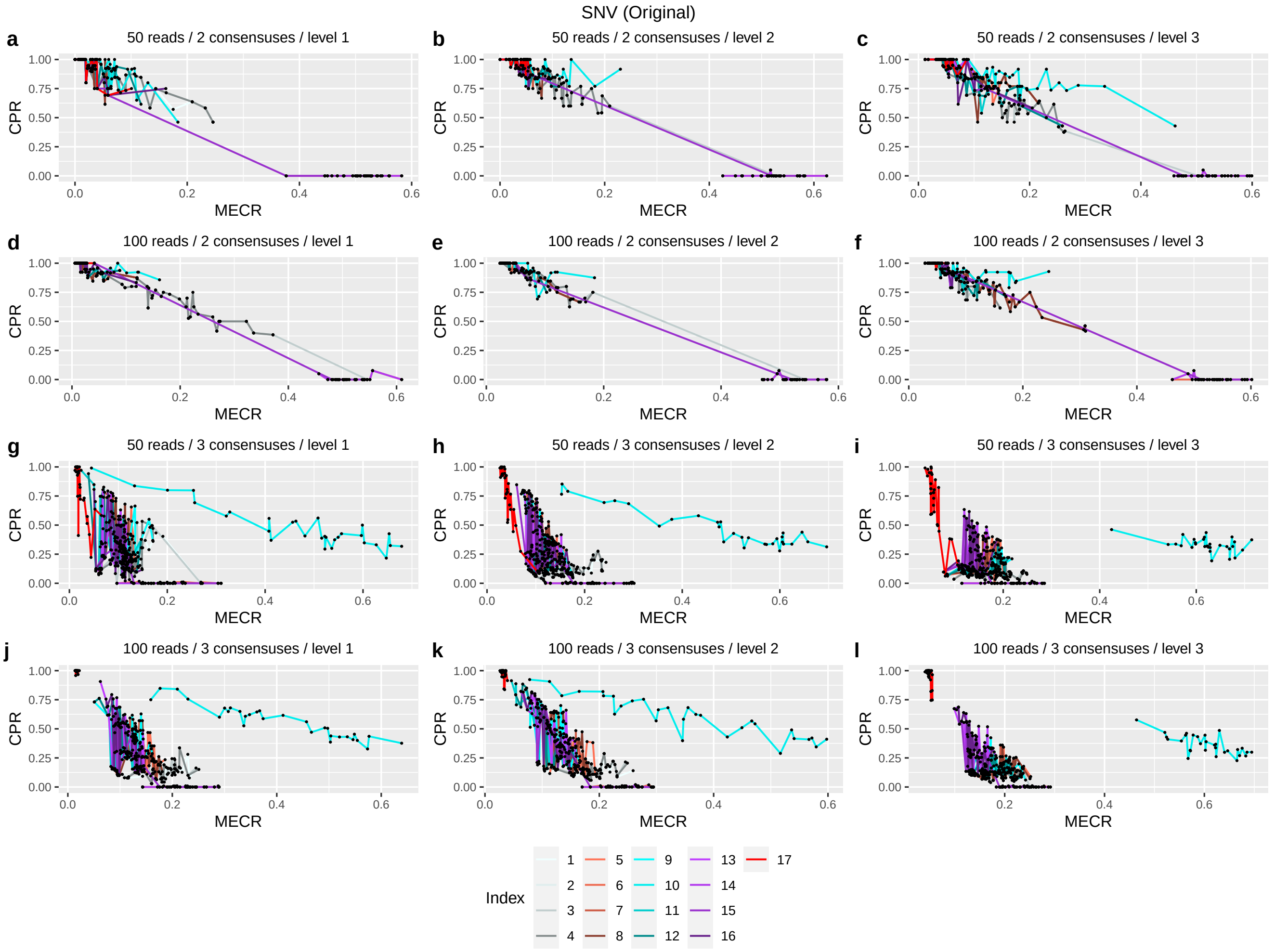

Figure S11: The negative relation between MECR score and CPR score in Simulation 1. All the methods show the negative relation among two indexes for 30 replications.

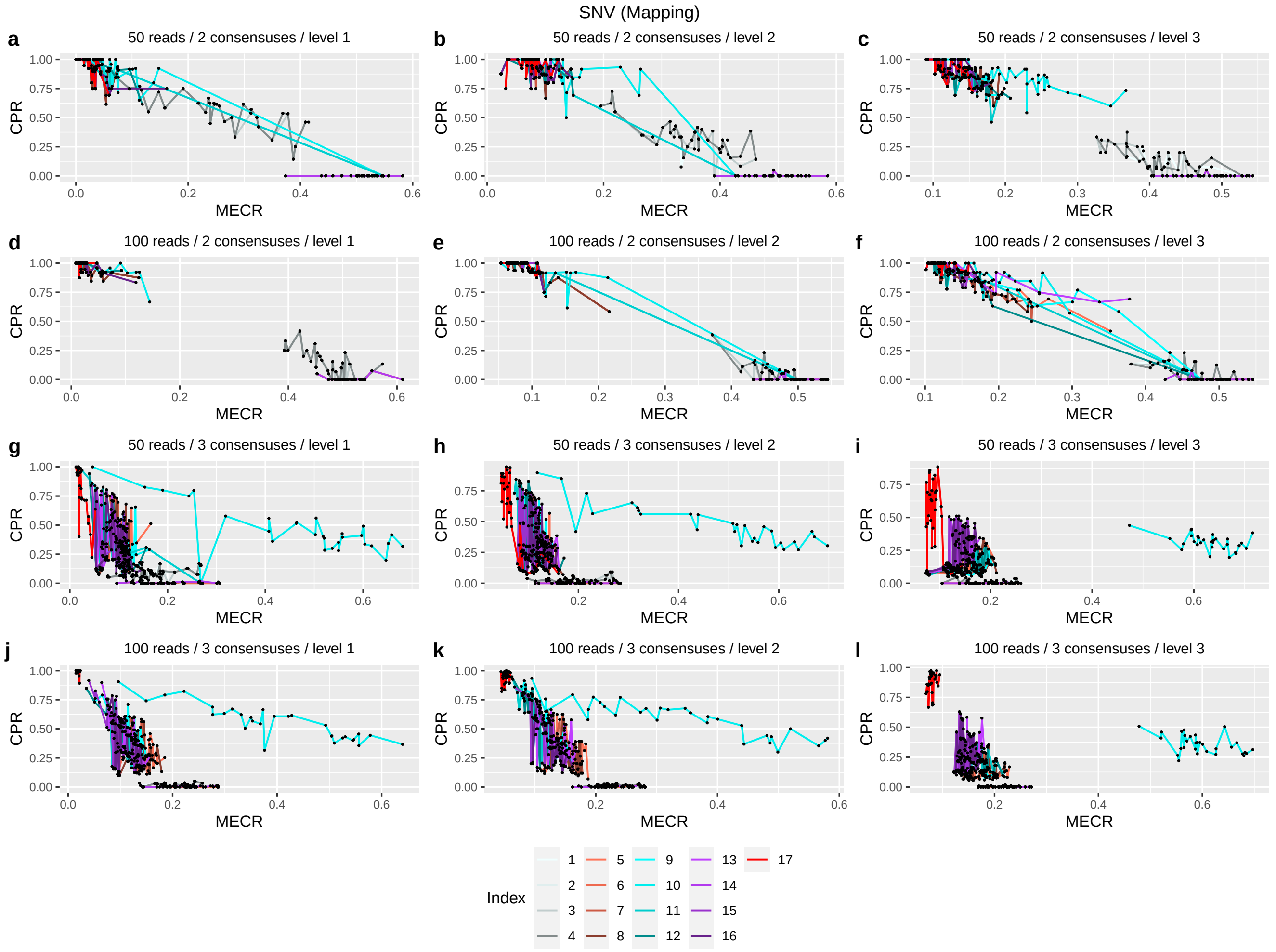

Figure S12: The negative relation between MECR score and CPR score in Simulation 2. All the methods show the negative relation among two indexes for 30 replications.

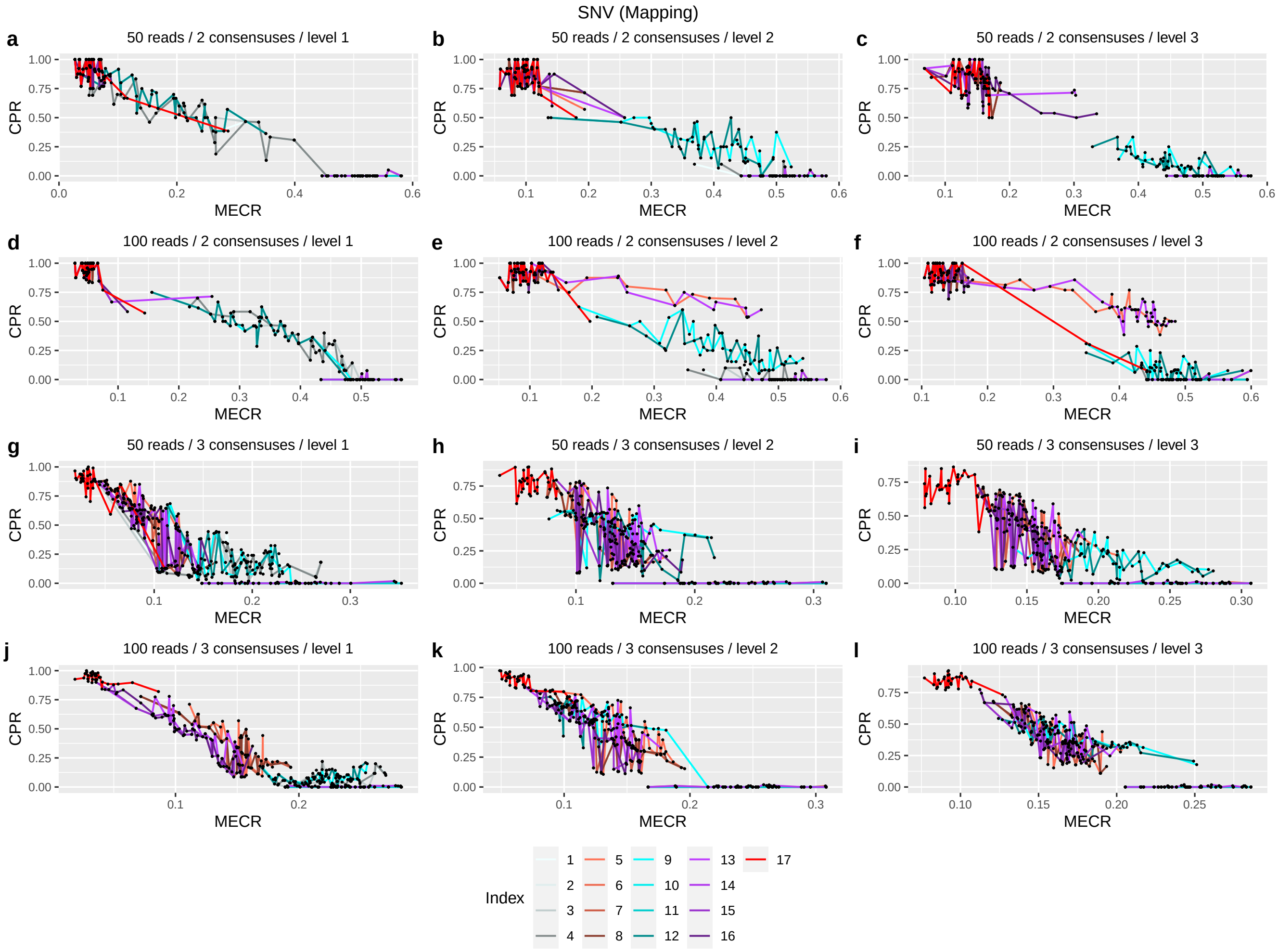

Figure S13: The negative relation between MECR score and CPR score in Simulation 3. All the methods show the negative relation among two indexes for 30 replications.

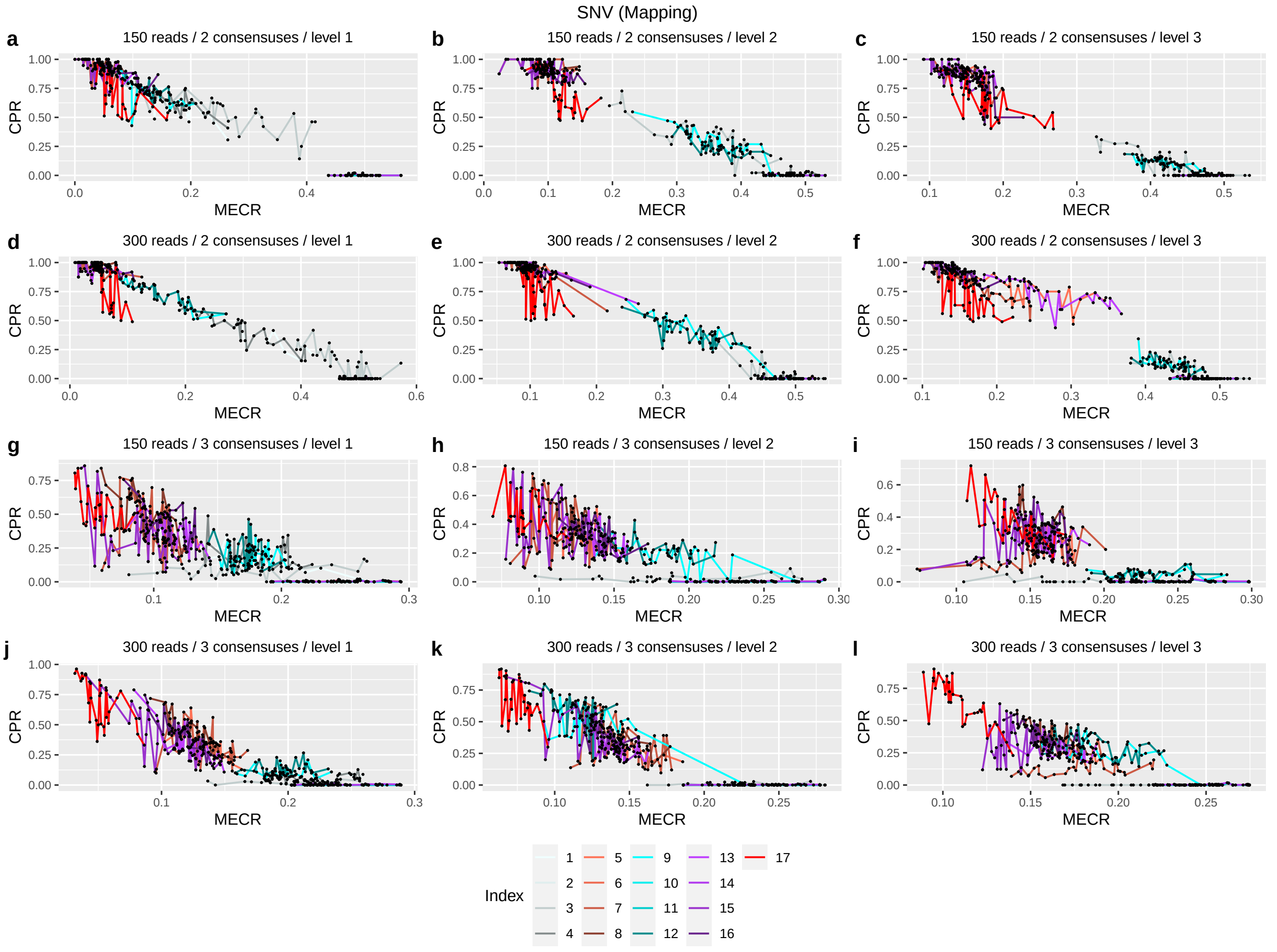

Figure S14: The negative relation between MECR score and CPR score in Simulation 4. All the methods show the negative relation among two indexes for 30 replications.

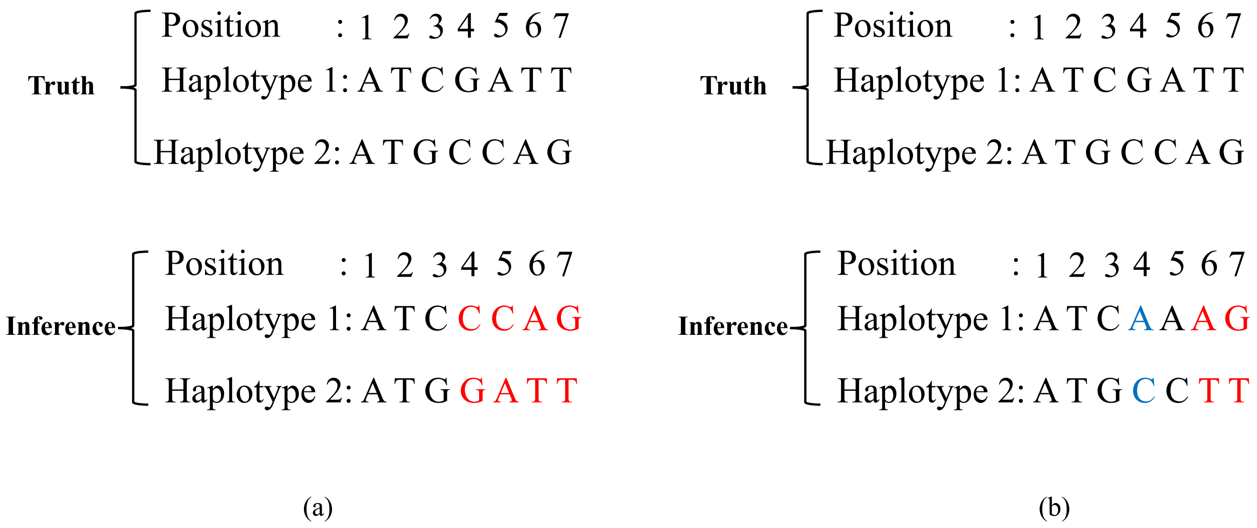

Figure S15: The toy example of switching error rate.(a) is the result of 1 switch between the 3rd and 4th SNV compared to the truth. (b) represents the example one wrong SNV in the inference result of 4th SNV and one switch between 5th and 6th SNV compared to the truth).

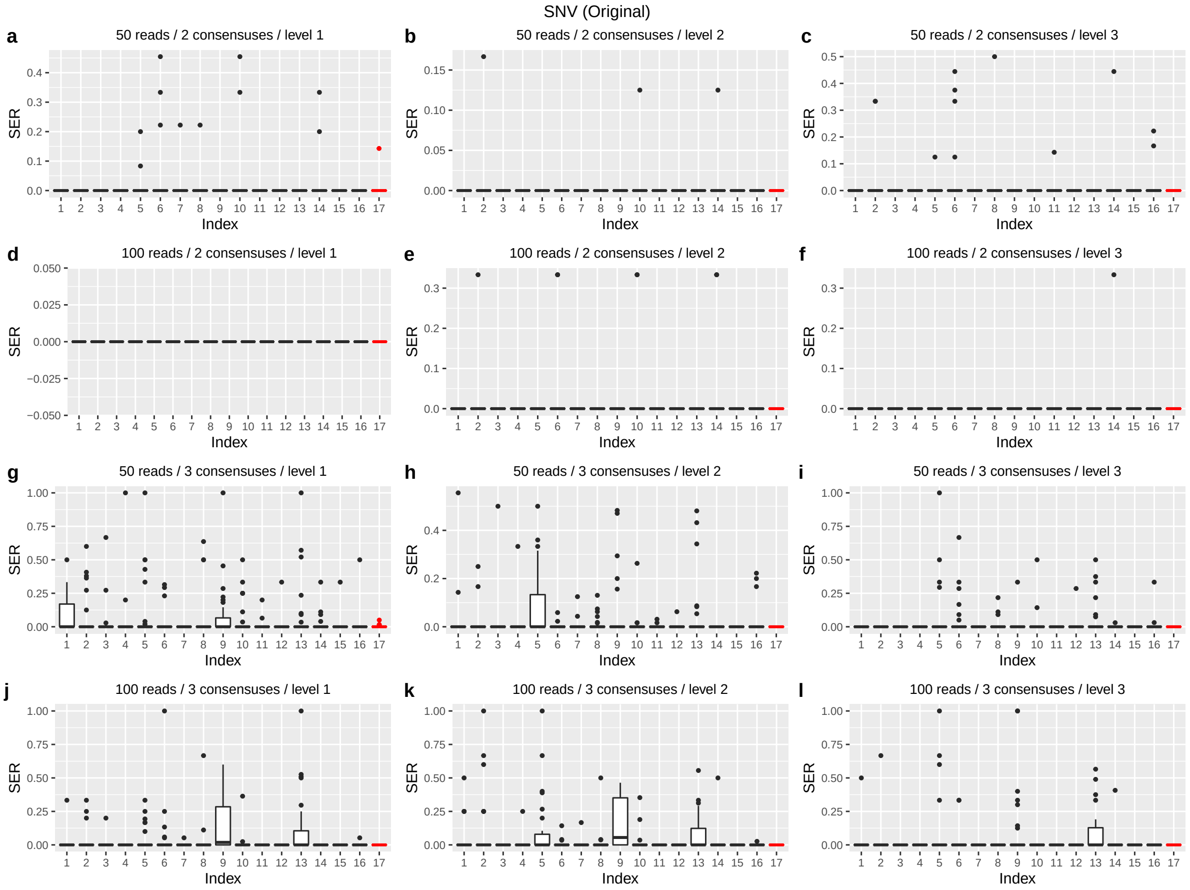

Figure S16: The switching error rate results in Simulation 1. In this simulation, we have the true mapping status of each read. *DIHap* almost phases all the SNVs in the 2 haplotypes (or consensus sequences) case and outperformed others in the 3 haplotypes case, especially in the higher coverage condition.

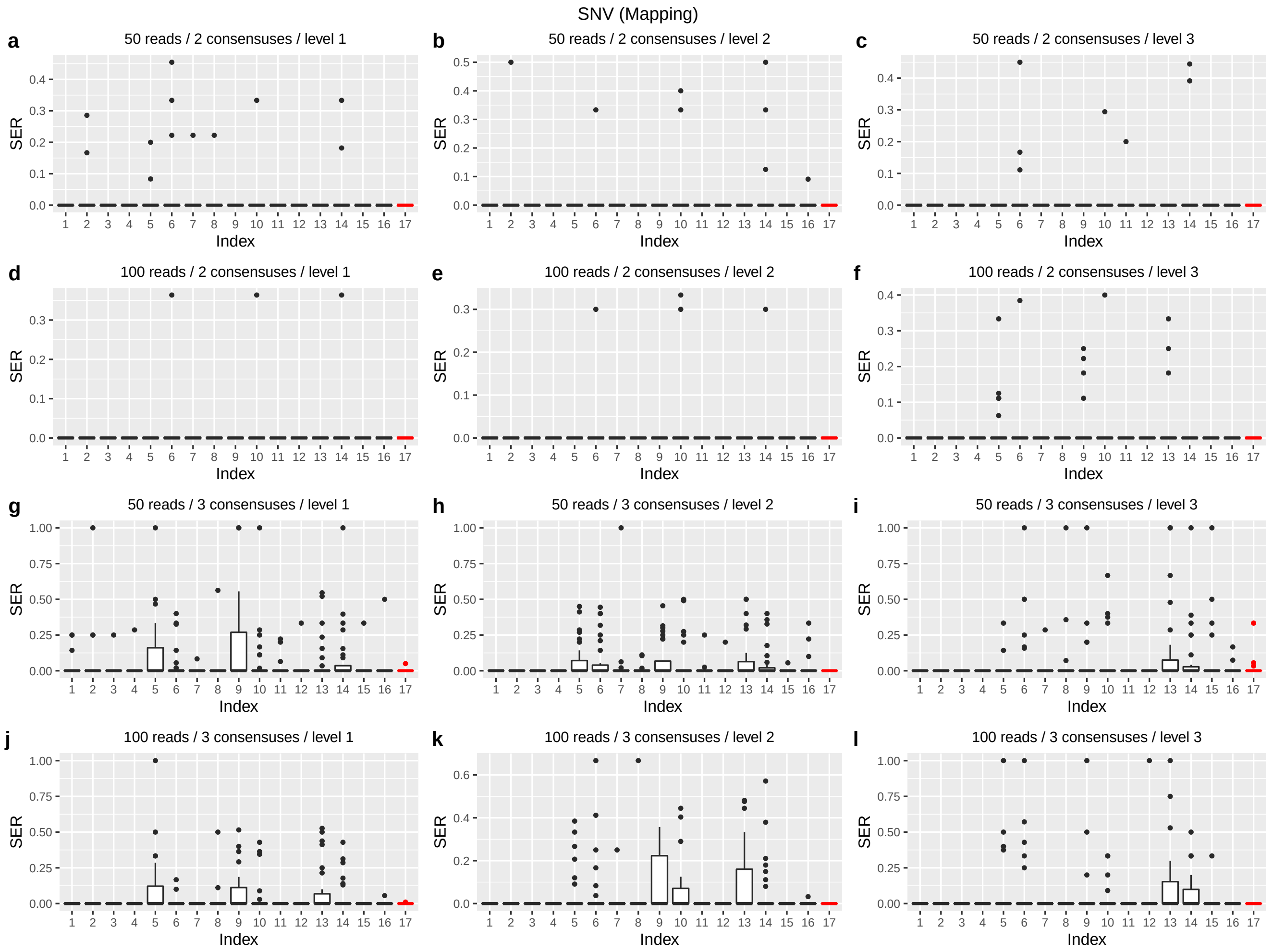

Figure S17: The switching error rate results in Simulation 2. In this simulation, we map the simulated reads to the reference sequence via *minimap2*. compared to Simulation 1, it further introduced the alignment error. *DIHap* still achieves a comparable performance in the 2 haplotypes (or consensus sequences) case and outperforms others in 3 haplotypes case.

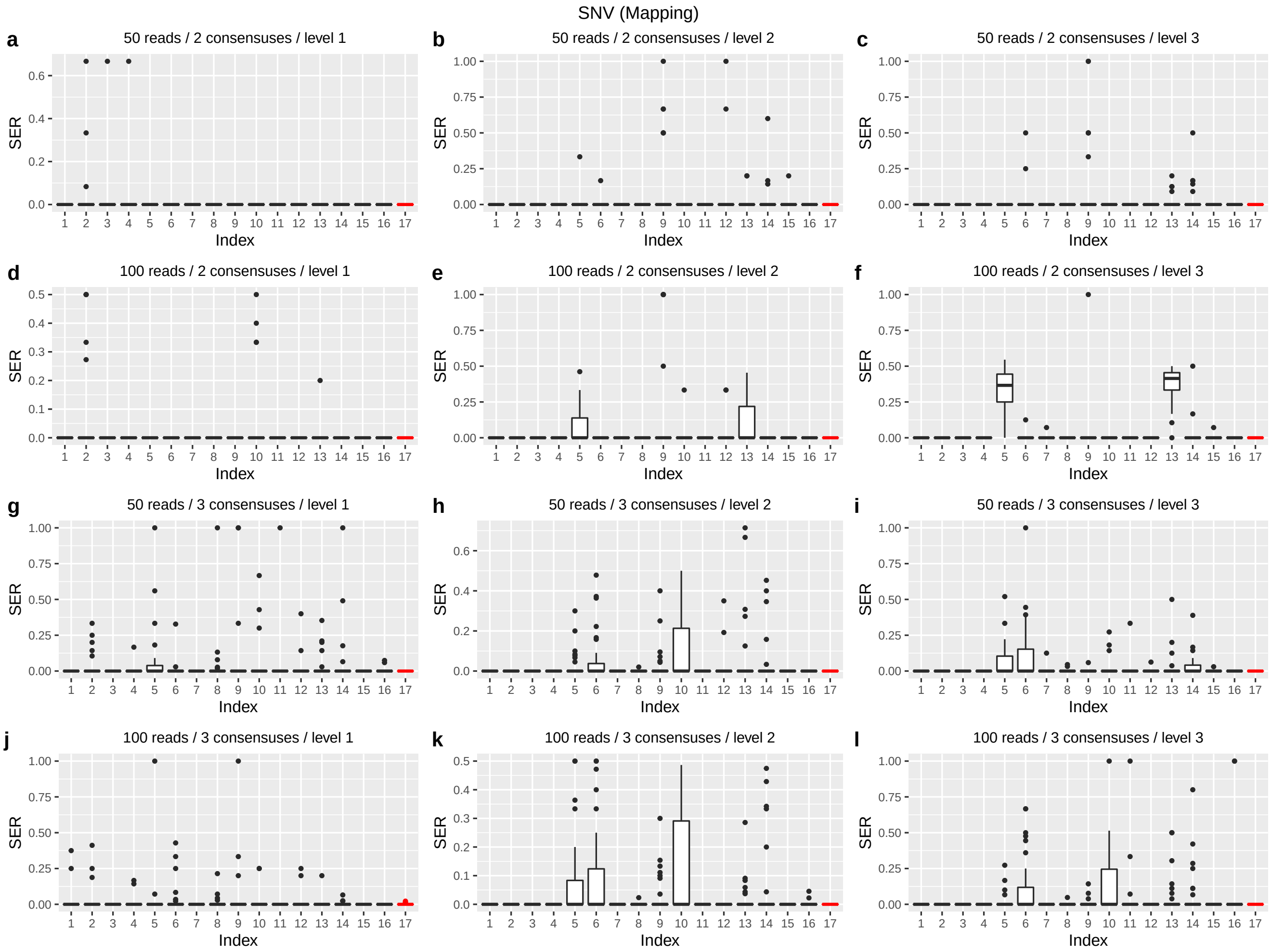

Figure S18: The switching error rate results in Simulation 3. Simulation 3 relies on *PbSim2* to simulate the reads based on the consensus sequences. *PbSim2* utilizes a different simulation procedure, representing a different sequencing error profile scenario. In this simulation, *DIHap* can obtain similar performance for diploidy data and achieve better results for polyploidy data.

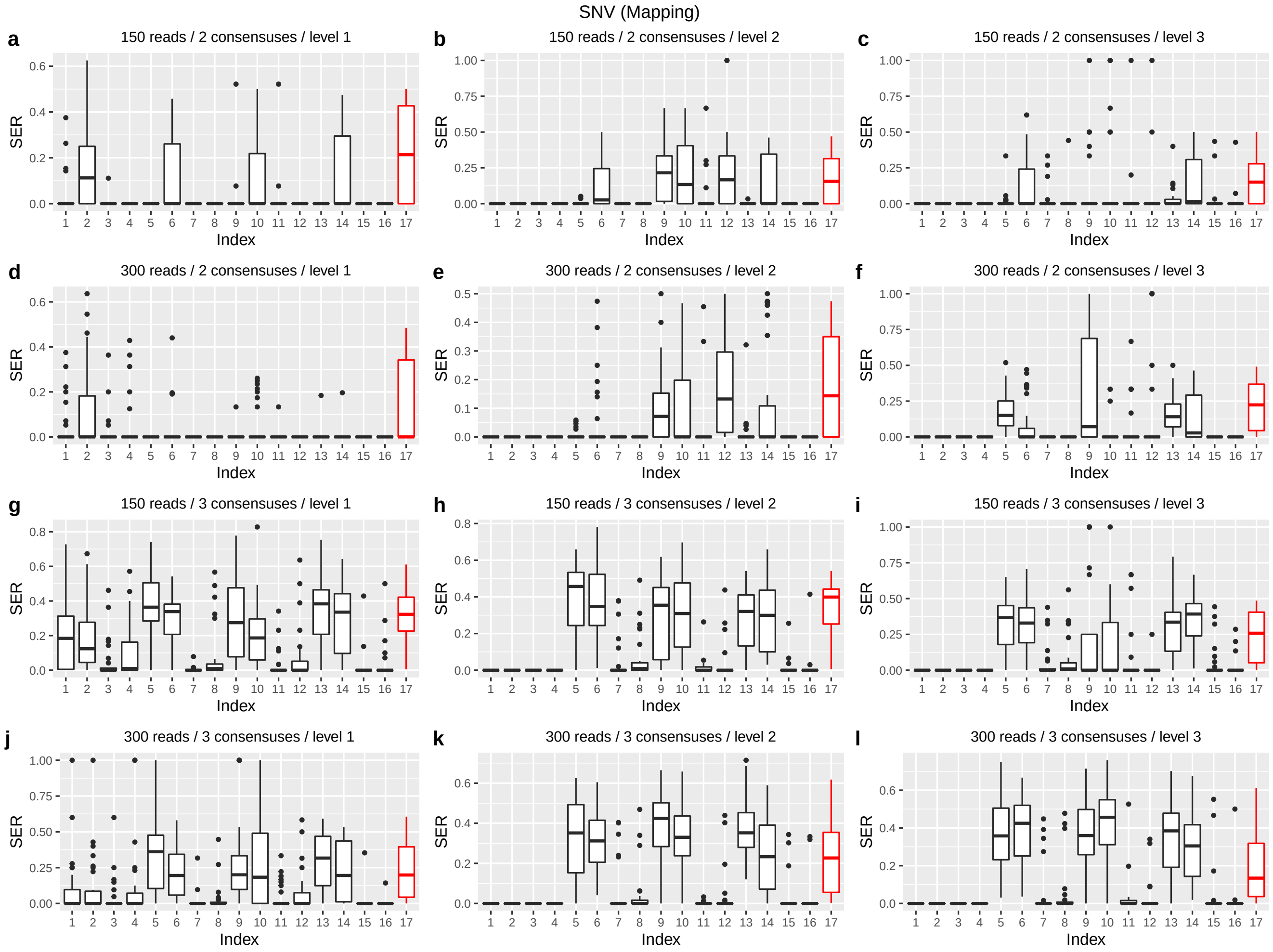

Figure S19: The switching error rate results in Simulation 4. Simulation 4 introduces structural variations to the consensus sequences, which can affect the detection of micro-variations due to biased mapping results. The SER of other baseline methods significantly increases in the $K = 3$ haplotypes (or consensus sequences); however, *DIHap* maintains a smaller SER.

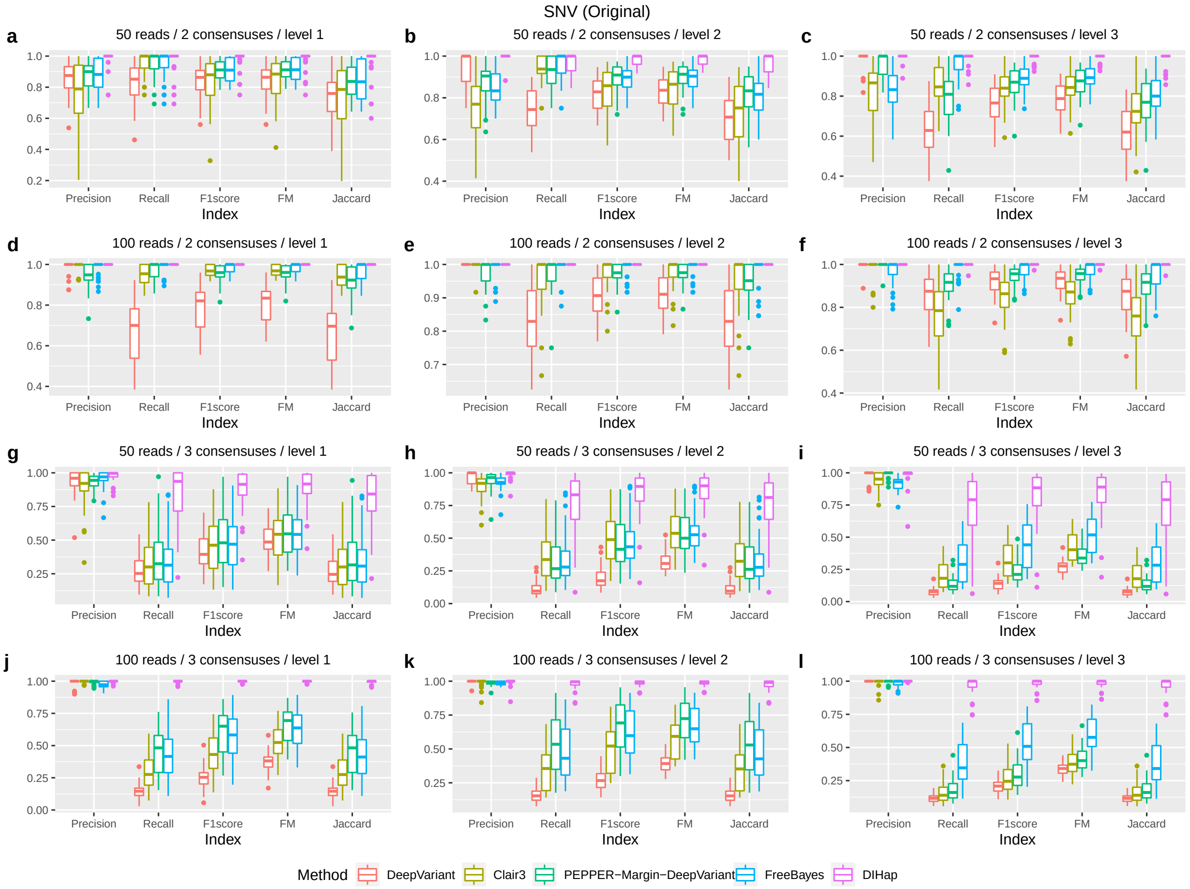

Figure S20: The results for SNV detection in Simulation 1. In this simulation, we have the true mapping status of each read. *DIHap* almost detected all the SNVs in the 2 haplotypes (or consensus sequences) case and outperformed others in the 3 haplotypes case, especially in the higher coverage condition.

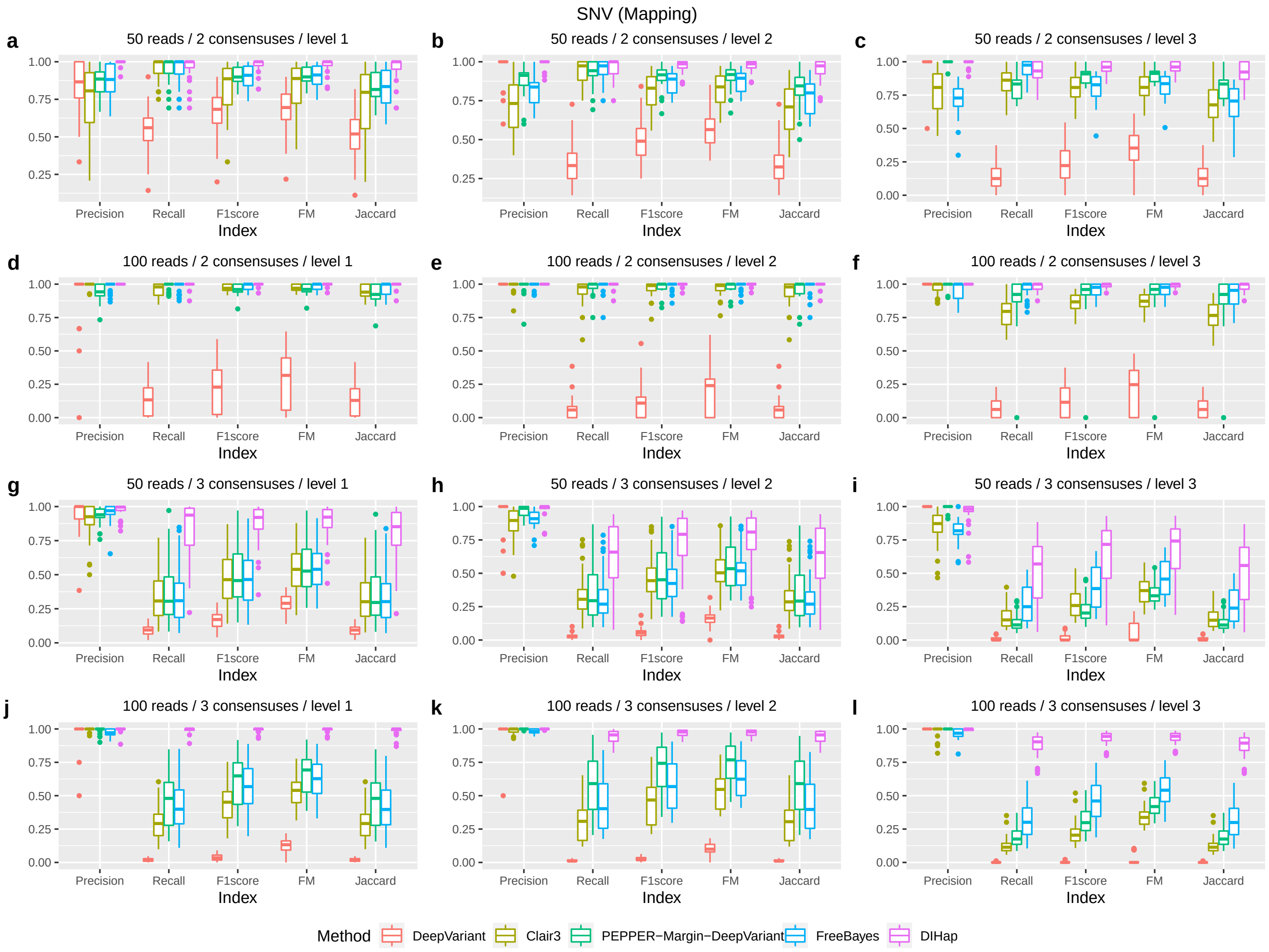

Figure S21: The results for SNV detection in Simulation 2. In this simulation, we map the simulated reads to the reference sequence via *minimap2*. compared to Simulation 1, it further introduced the alignment error. *DIHap* still achieves a comparable performance in the 2 haplotypes (or consensus sequences) case and outperforms others in 3 haplotypes case.

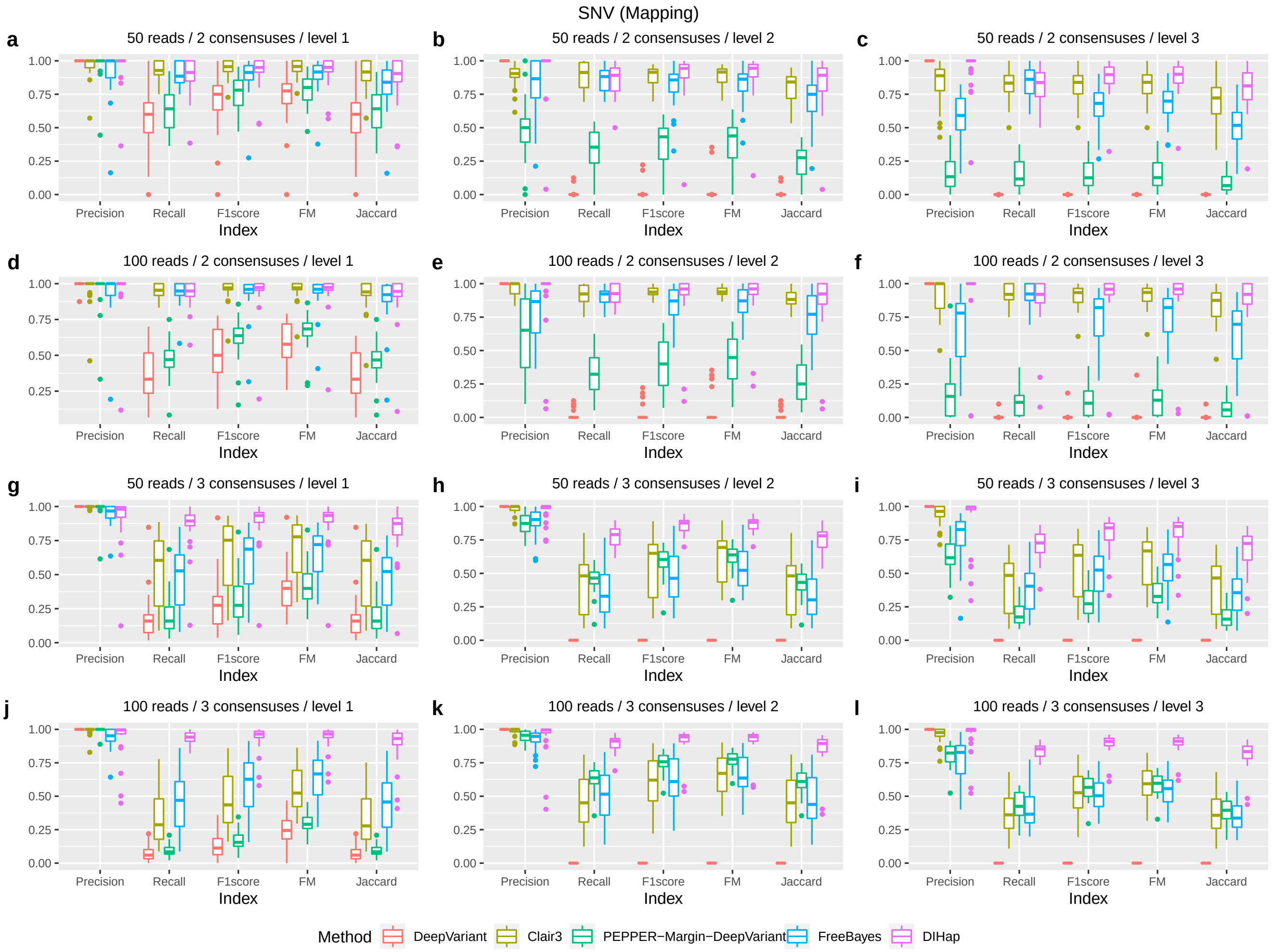

Figure S22: The results for SNV detection in Simulation 3. Simulation 3 relies on *PbSim2* to simulate the reads based on the consensus sequences. *PbSim2* utilizes a different simulation procedure, representing a different sequencing error profile scenario. In this simulation, *DIHap* can obtain similar performance for diploidy data and achieve better results for polyploidy data.

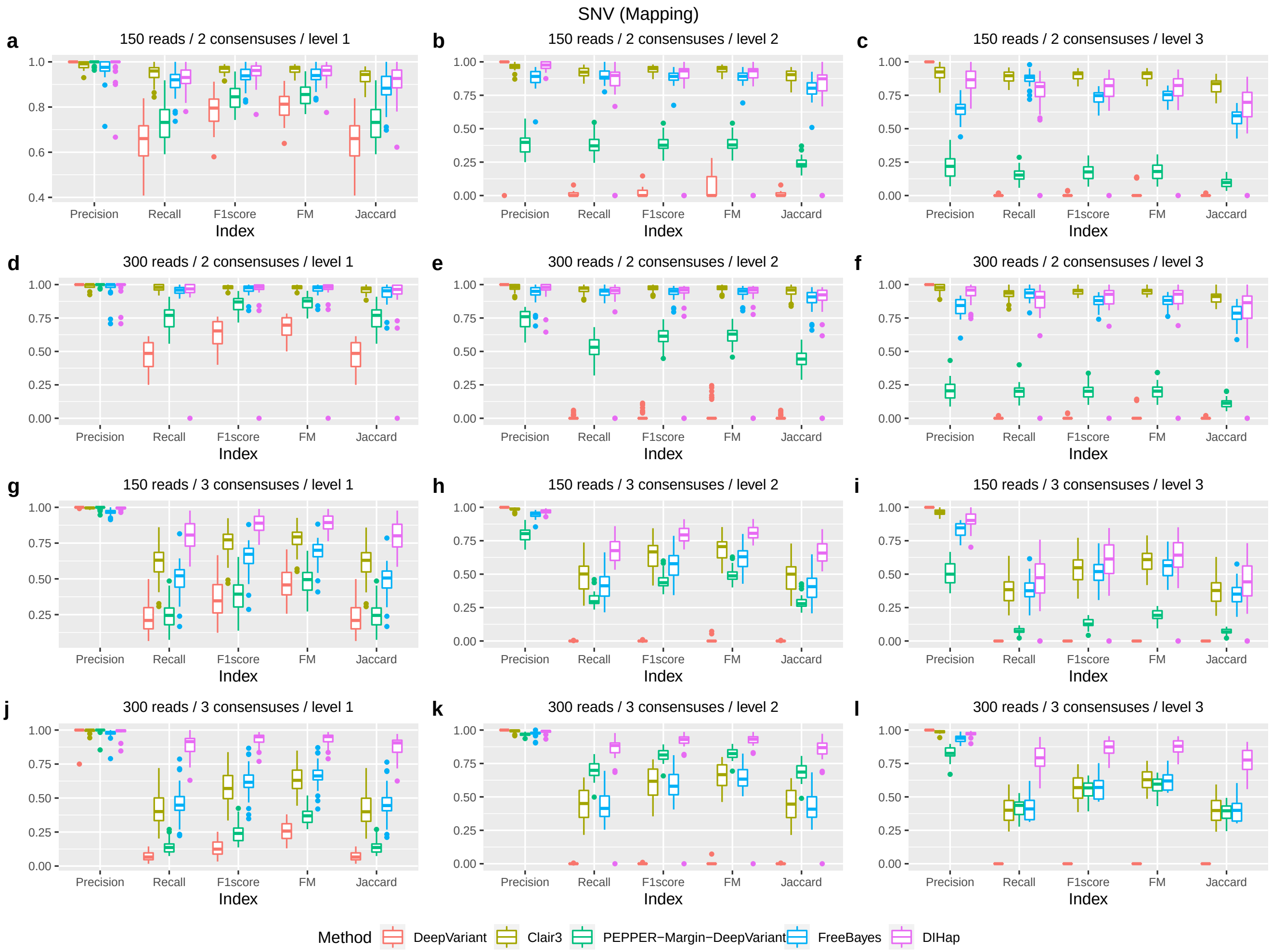

Figure S23: The results for SNV detection in Simulation 4. Simulation 4 introduces structural variations to the consensus sequences, which can affect the detection of micro-variations due to biased mapping results. The recall of other baseline methods significantly declines in the $K = 3$ haplotypes (or consensus sequences); however, *DIHap* maintains a higher recall value.

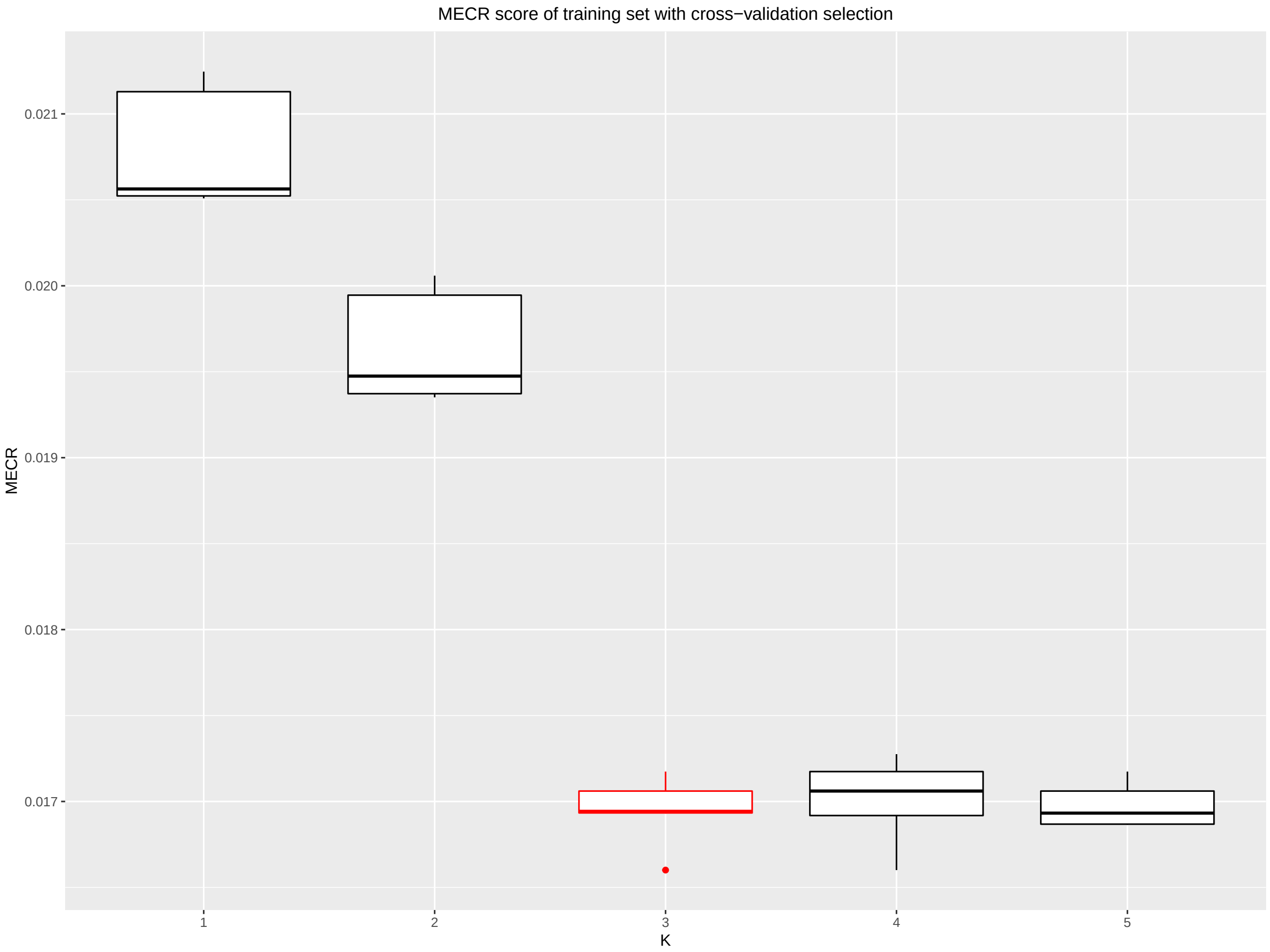

Figure S24: One example of selecting K used a 5-fold cross-validation scheme. Red color represents the truth. Each boxplot illustrates the distribution of the MECR scores for the validation set. The MECR score decreases as the number of haplotypes increases, with $K = 3$ identified as the elbow point.

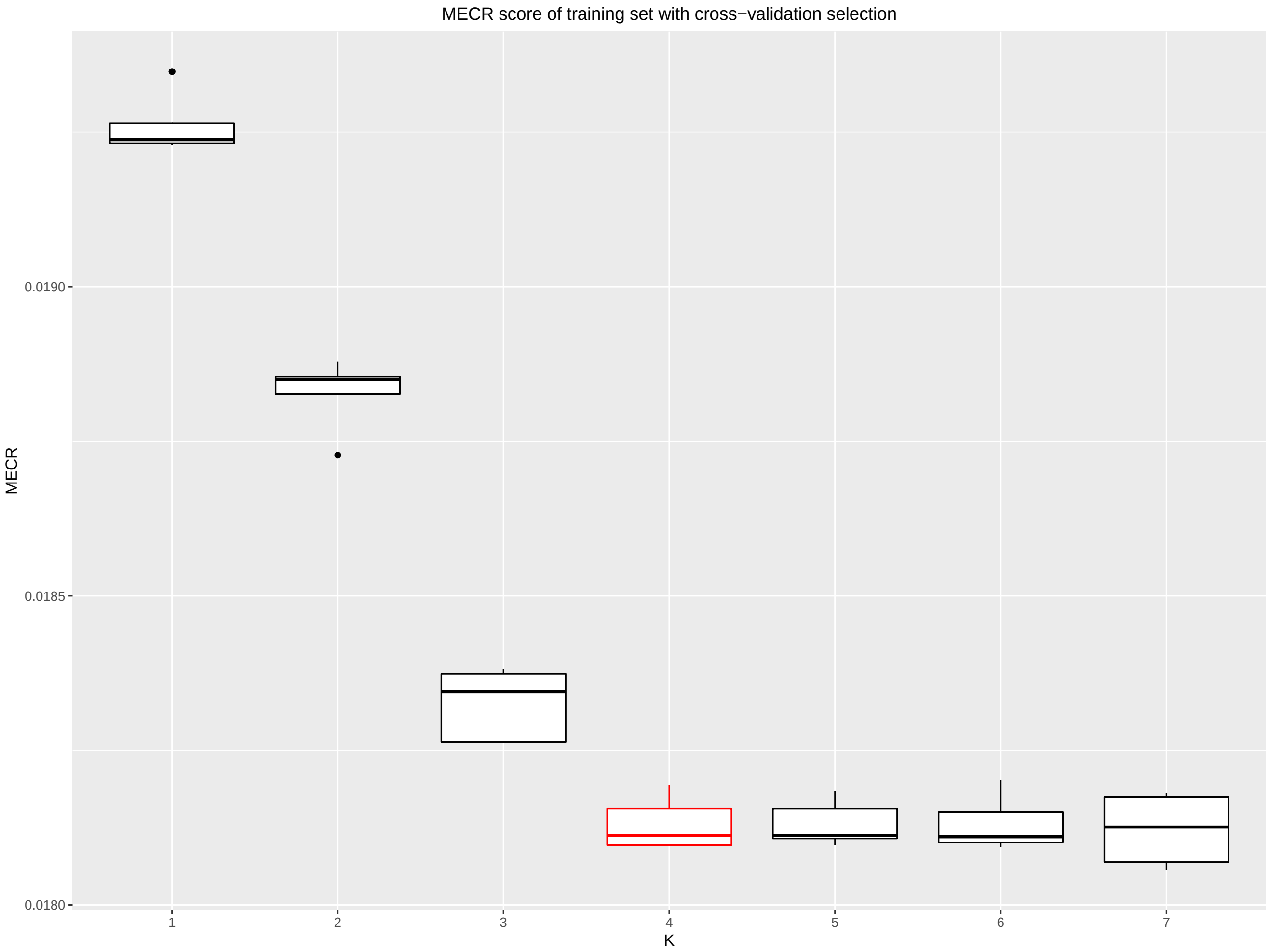

Figure S25: Another example of selecting K used a 5-fold cross-validation scheme. Red color represents the truth. Each boxplot illustrates the distribution of the MECR scores for the validation set. The MECR score decreases as the number of haplotypes increases, with $K = 4$ identified as the elbow point.

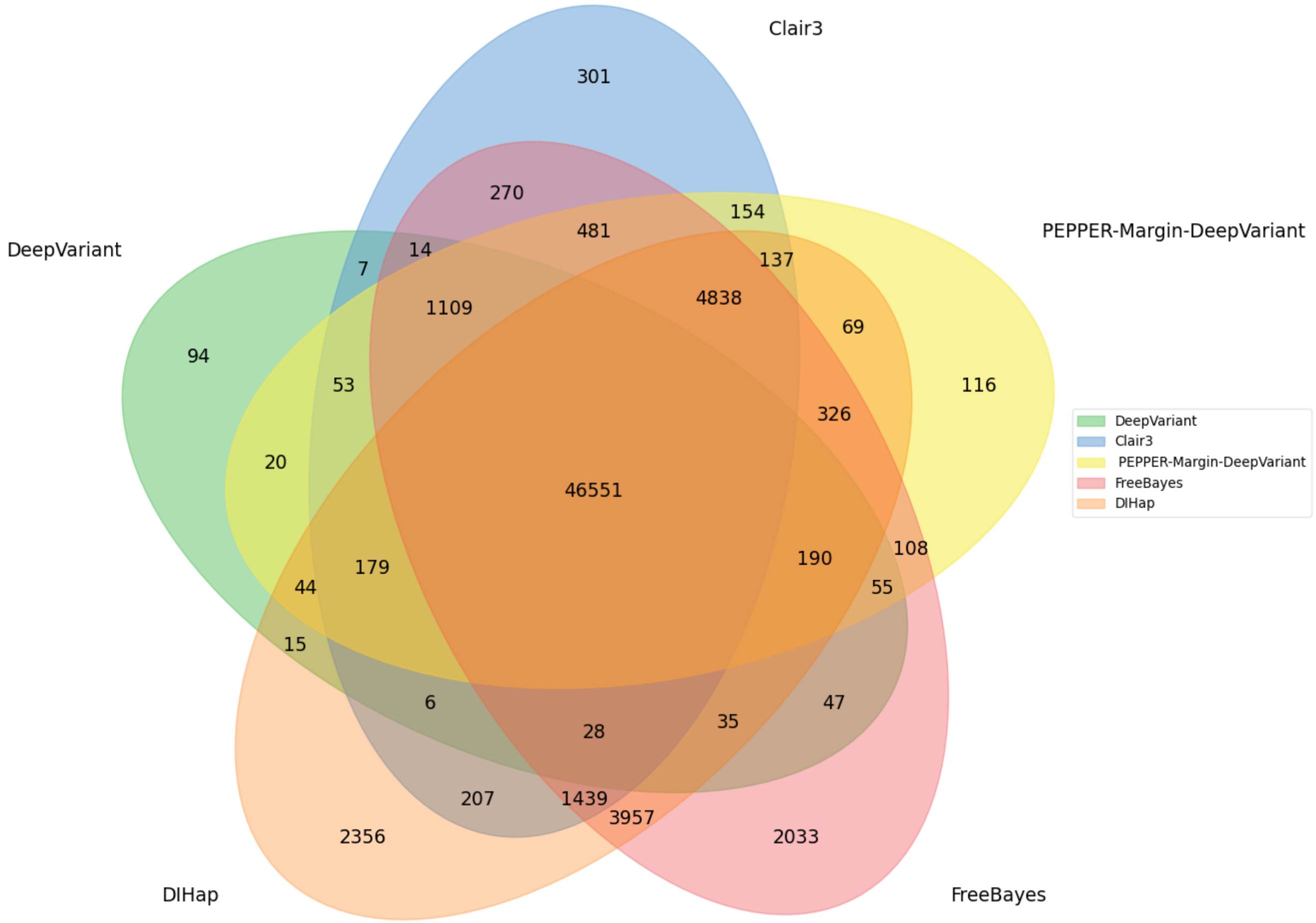

Figure S26: Venn plot of the detection results of algorithms of HG002.

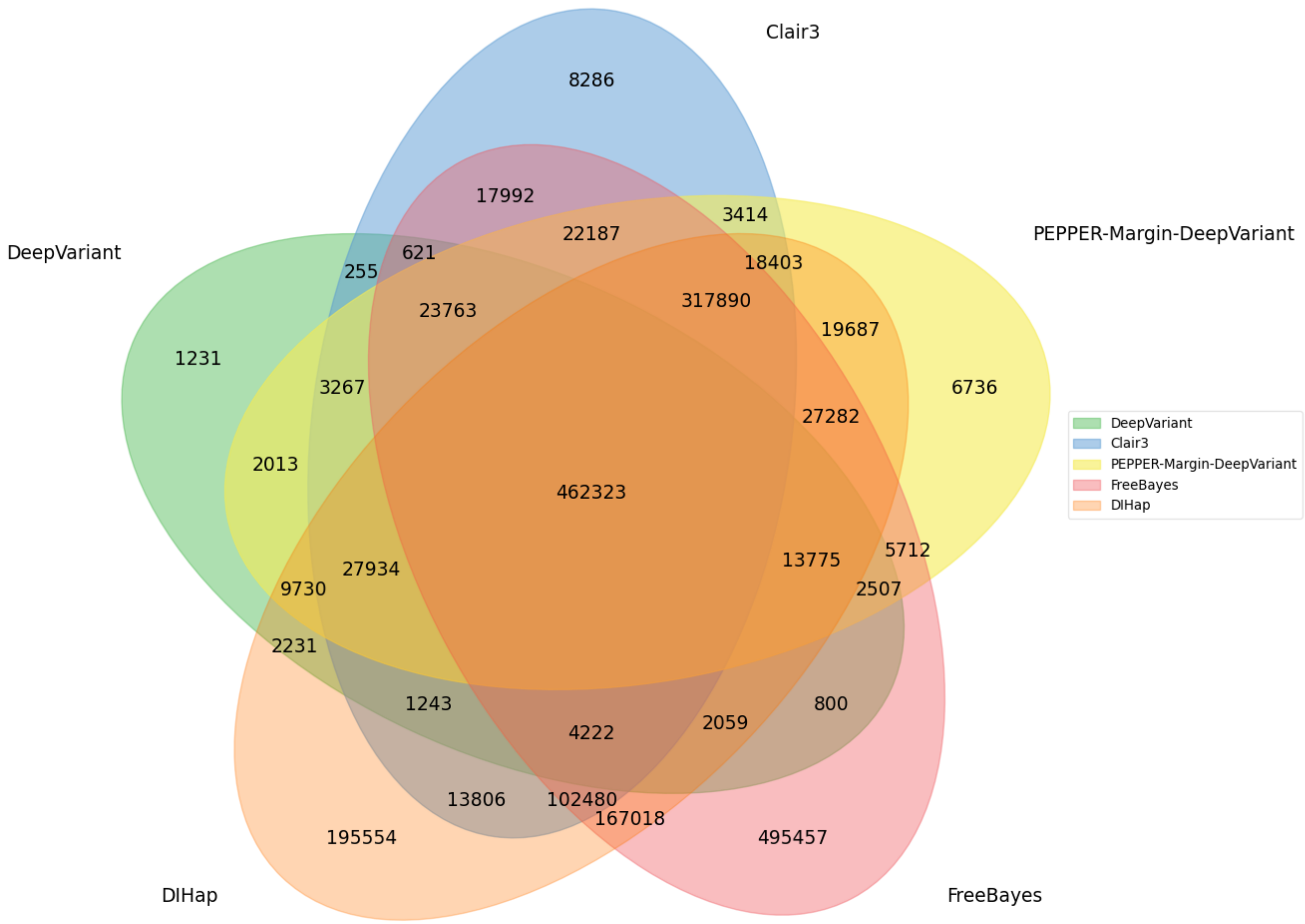

Figure S27: Venn plot of the detection results of algorithms of Otava.

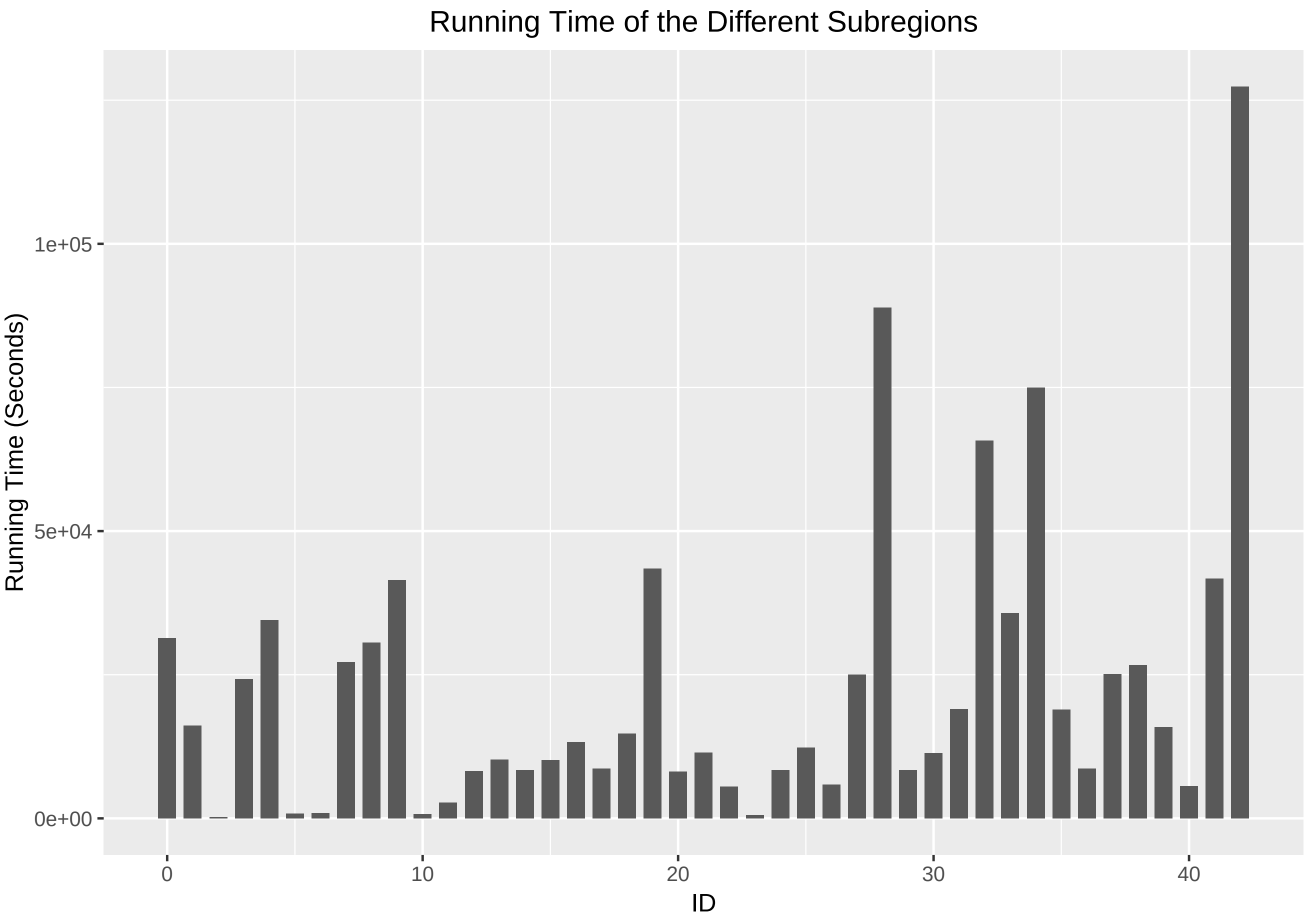

Figure S28: Running Time of different subregions of HG002 chromosome 22.

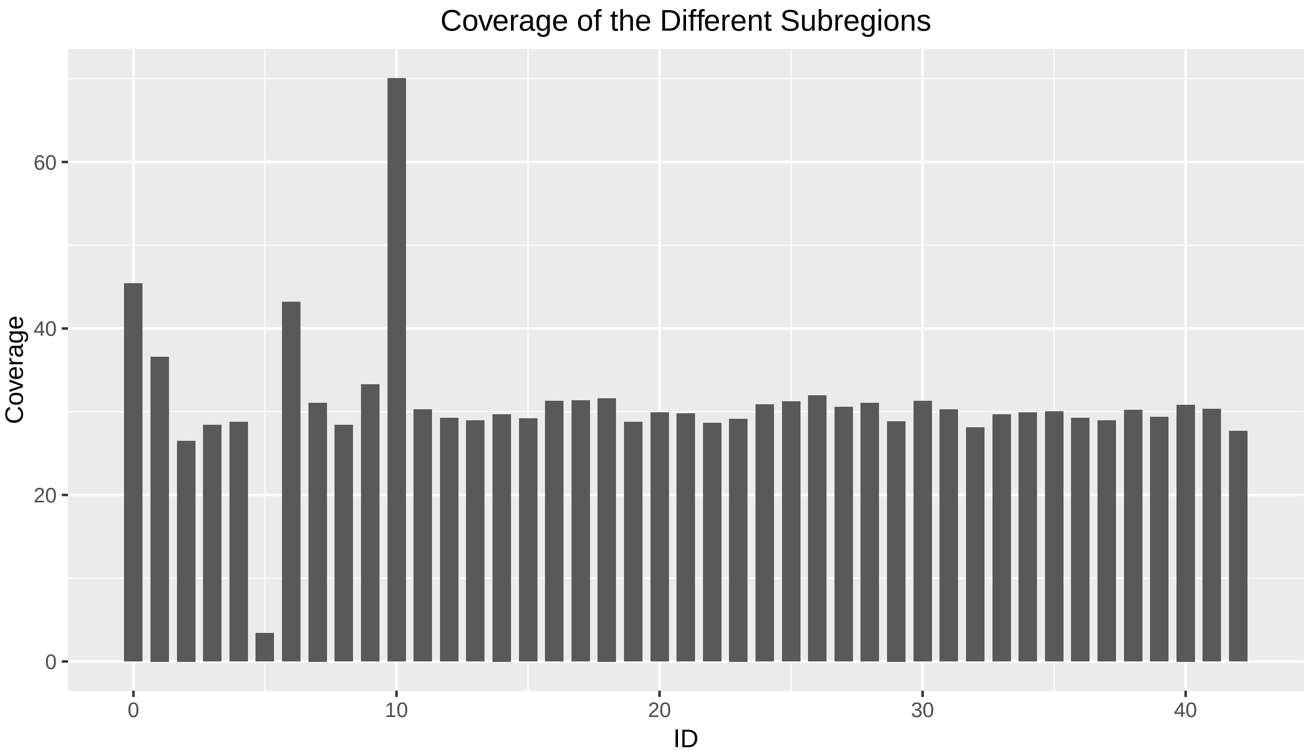

Figure S29: Coverage of the different Subregions of HG002 chromosome 22.

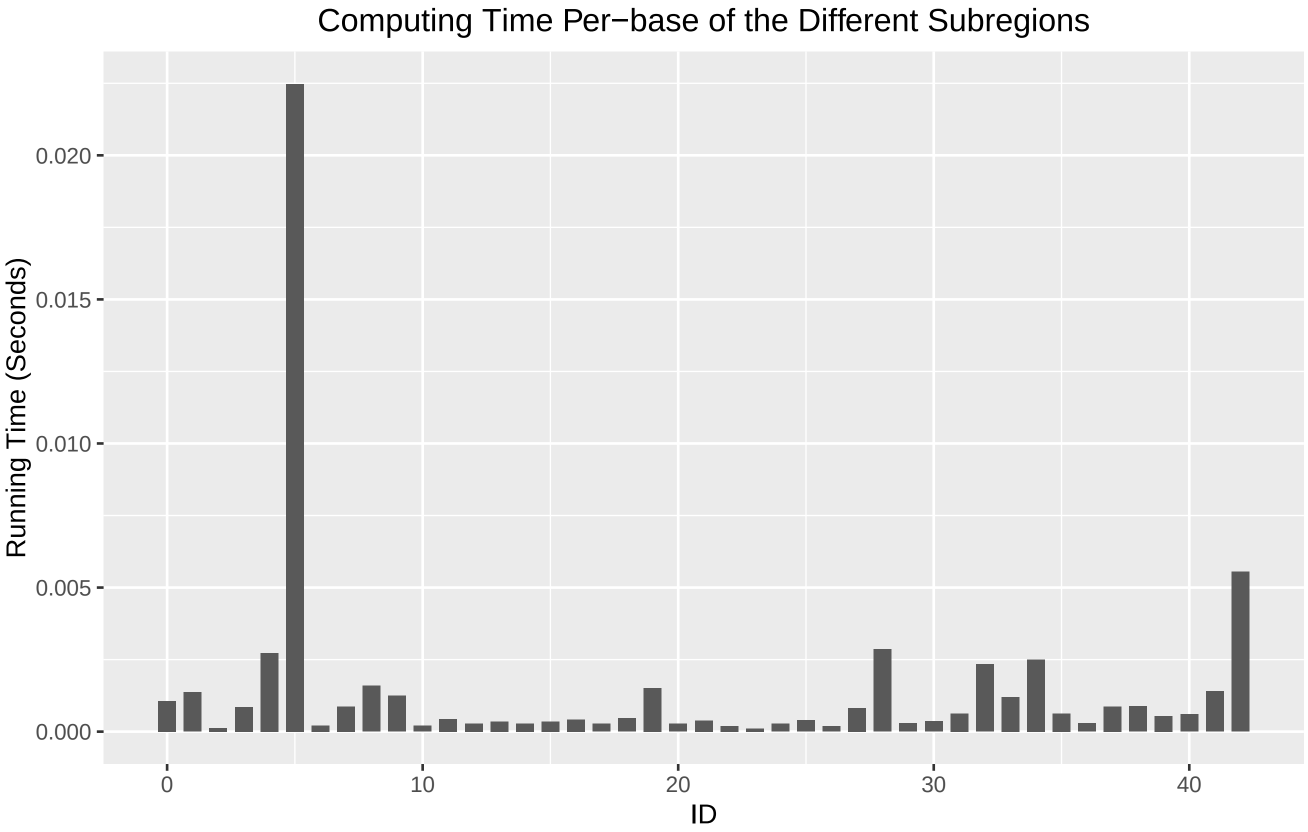

Figure S30: Computing Time Per-base of the Different Subregions of HG002 chromosome 22.
